# Community recovery dynamics in yellow perch microbiome after gradual and constant metallic perturbations

**DOI:** 10.1186/s40168-020-0789-0

**Published:** 2020-02-10

**Authors:** Bachar Cheaib, Hamza Seghouani, Umer Zeeshan Ijaz, Nicolas Derome

**Affiliations:** 1 Institut de Biologie Intégrative et des Systèmes (IBIS), Pavillon Charles-Eugène Marchand, Université Laval, 1030, avenue de la Médecine, Québec, QC G1V 0A6 Canada; 2 School of Engineering, University of Glasgow, Glasgow, G12 8QQ Scotland

**Keywords:** Fish microbiome, Disturbance, Recovery, Stress gradient, Neutrality, Evolutionary forces, Community assembly, Pathogens, Metagenomics

## Abstract

**Background:**

The eco-evolutionary processes ruling post-disturbance microbial assembly remain poorly studied, particularly in host-microbiome systems. The community recovery depends not only on the type, duration, intensity, and gradient of disturbance, but also on the initial community structure, phylogenetic composition, legacy, and habitat (soil, water, host). In this study, yellow perch (*Perca flavescens*) juveniles were exposed over 90 days to constant and gradual sublethal doses of cadmium chloride. Afterward, the exposure of aquaria tank system to cadmium was ceased for 60 days. The skin, gut and water tank microbiomes in control and treatment groups, were characterized before, during and after the cadmium exposure using 16s rDNA libraries and high throughput sequencing technology (Illumina, Miseq).

**Results:**

Our data exhibited long-term bioaccumulation of cadmium salts in the liver even after two months since ceasing the exposure. The gradient of cadmium disturbance had differential effects on the perch microbiota recovery, including increases in evenness, taxonomic composition shifts, as well as functional and phylogenetic divergence. The perch microbiome reached an alternative stable state in the skin and nearly complete recovery trajectories in the gut communities. The recovery of skin communities showed a significant proliferation of opportunistic fish pathogens (i.e., *Flavobacterium*). Our findings provide evidence that neutral processes were a much more significant contributor to microbial community turnover in control treatments than in those treated with cadmium, suggesting the role of selective processes in driving community recovery.

**Conclusions:**

The short-term metallic disturbance of fish development has important long-term implications for host health. The recovery of microbial communities after metallic exposure depends on the magnitude of exposure (constant, gradual), and the nature of the ecological niche (water, skin, and gut). The skin and gut microbiota of fish exposed to constant concentrations of cadmium (CC) were closer to the control negative than those exposed to the gradual concentrations (CV). Overall, our results show that the microbial assembly during the community recovery were both orchestrated by neutral and deterministic processes.

**Video Abtract.**

## Background

Resilience refers to the capacity of a natural ecosystem to maintain a stable state after facing different exogenous disturbances, both in terms of amplitude and frequency [1]. Introduced first by Holling (1973), the concept of resilience was redefined to incorporate the idea of recovery following a temporary disruption [2, 3], not simply the ability to resist this disturbance in the first place [4]. Both ecological concepts, “resistance” and “recovery,” were simultaneously considered as measurable components that together represent resilience [4]. In other microbial studies, the term “resistance” is synonymous with resilience [5] using Holling‘s definition. Notwithstanding, “sensitivity” (inverse of resistance) is also sometimes used to represent the degree to which a community changes in response to disturbance [6]. The recovery rate, time to reach an equilibrium state, and the distance to an alternate stable state are quantitative measures that can be used to compare the resilienc e[4, 7–9] and improve our understanding of ecosystem recovery [6, 10]. In this study, we will employ the term “recovery” to describe the pattern of eco-evolutionary change that occurs when a community returns to an alternative stable state.

The recovery of microbial communities depends on the type, duration, intensity, and variability of a disturbance. More importantly, microbial recovery can be impacted by the initial community structure, phylogenetic composition, legacy, and the type of habitat (soil, water, host). After antibiotic treatment, the complete recovery of initial bacterial community composition is rarely achieved, as reported in various host-microbiota systems from honeybees [11] to humans [12]. The incomplete recovery of gut microbiota ecosystems after antibiotic administration results in a shift of the microbial composition to an alternative equilibrium called an “alternate stable state” [6, 13, 14]. This compositional shift occurs when resistance or recovery is weak and/or when the intensity of disturbance is high. Although the understanding of factors that drive such regime shifts to an alternative equilibrium in microbial ecosystems will have tremendous impacts in various fields of application (e.g., personalized medicine, agriculture, bioremediation), this phenomenon is still poorly studied.

The relative roles of ecological and evolutionary processes in the recovery of the structure of microbial communities are still to decipher. Theoretically, the nature of these processes can be neutral (stochastic) [15, 16], or selective (deterministic) [17, 18], the latter being driven either by environmental filtering or competitive exclusion [19, 20], the former by demographic sampling effects alone. In the context of community recovery, a small number of studies revealed that deterministic processes drive bacterial succession dynamics in a soil bacterial community disrupted either by a depletion gradient of nutrients [21], a thermal shock [22], or a rainfall rehydration of dry soil [23].

In the present study, we assessed the relative contribution of neutral and deterministic processes in the recovery of the yellow perch (*Perca flavescens*) microbiome assembly following an experimental metallic exposure gradient. Polymetallic contamination in aquatic ecosystems mostly results from exposure to acid mine drainages (AMD) occurring around the world [24–34]. For instance, in the natural Canadian lakes, the cadmium (Cd) concentration reaches 9 ppb (parts per billion) in perch liver/water [35, 36], and it has a clear quantitative impact on the perch physiology, gene expression, and genotype diversity [37]. In the same polluted lake system studied by Couture et al. (2008), the microbial communities’ assembly in water has evolved under chronic exposure to a gradient of trace metals due to the AMD expelled in the environment, leaving substantial genotypic signatures of adaption in the taxonomic and functional repertories of AMD communities [34]. Given that yellow perch juveniles can tolerate sublethal doses of cadmium without encountering significant physiological damage or death [38, 39], this host-microbiota model system is well suited to study microbiota recovery following metal exposure stress. In the laboratory, yellow perch juveniles underwent exposure to sublethal doses of cadmium chloride (CdCl_2_), the accumulation of which was tested in the water and within perch liver. The recovery of community structure and function in water and host microbiome were then studied and compared between constant and variable regimes of metallic stress, which was defined by the levels of Cd detected in liver and water samples. To disentangle the effect of the xenobiotic from host development [40, 41] on bacterial strain recruitment ontogeny, microbiota assembly was also assessed in stable conditions as a control regime. Our expectation was that constant exposure to cadmium chloride, due to its severe implications for host and microbial community physiology, would impede community recovery most severely than in the gradual exposure experimental group.

## Methods

### Fish rearing

**Fig. 1:**
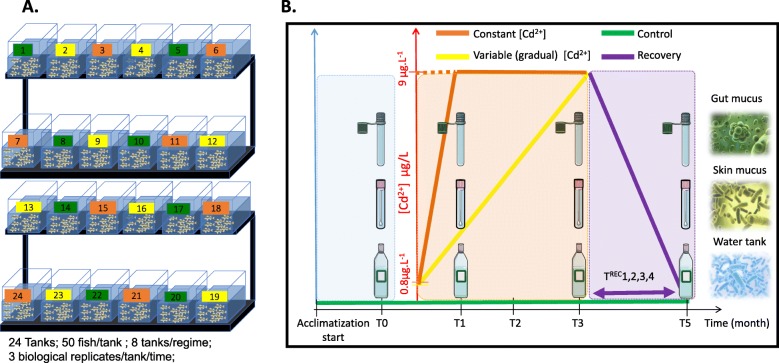
Schematic illustration of the perch microbiome recovery experiment

The experiment is described in Fig. 1: Schema 1. Briefly, there were two acclimation periods: one in a standard container (1500L) and the second in 24 tanks (36 L) with an independent filtering system circuit for each aquarium. The fish juveniles were reared within the same physicochemical conditions (photoperiod, pH, ammonia, nitrogen dioxide). Throughout the experimental period, to maintain viable conditions for perch in each water tank, fecal and uneaten food particles were removed daily using specific pressing tubes for each set of experimental conditions. A volume of 15 L of water was renewed two times a week for each tank (Fig. 1:Schema 1).

### Exposure regimes to cadmium

Cd-treated and control (Ctrl) tanks were designed into two cadmium chloride exposure regimes (8 tanks per regime), and one negative control regime (8 tanks) (Fig. 1: Schema 1). The yellow perch in treated tanks were exposed to cadmium chloride (CdCl_2_) dissolved in water. Under the regime of constant CdCl_2_ concentration exposure (CC), the cadmium chloride was initially added at 0.8 ppb, then increased to reach a target theoretical concentration of 9 ppb (parts per billion) by the end of the first month (T1). The CdCl_2_ concentration was adjusted to 9 ppb every 5 days during two additional months until the end of treatment (third month, T3) where the measured concentration reached an average of 5.8 ppb. Under the regime of variable CdCl_2_ concentration (CV), the CdCl_2_ was initially added at 0.6 ppb, then the concentration was gradually increased every 5 days to meet the target theoretical concentration of 9 ppb by the end of the third month. The measured concentration reached an average of 6.8 ppb at the end of treatment (third month, T3). The maximal CdCl_2_ concentration was settled at 9 μg/mL, which was within range of concentrations detected in yellow perch liver in contaminated Canadian lakes [35, 36].

### Recovery after the exposure to cadmium

The cadmium administration was stopped after the third month (T3). The experiment was extended 2 months (T5) after T3 to test the recovery of microbiome assembly in water and host.

### Host-microbiota and water sampling

Briefly, we selected 144 mucosa samples of skin (2 times × 3 regimes × 8 tanks × 3 replicates) and 144 gut (2 times × 3 regimes × 8 tanks × 3 replicates) samples corresponding to T0 (no cadmium) and T3 (ultima cadmium treatment). Also, 48 water samples (2 times × 3 regimes × 8 tanks × 1 technical replicate) from T0 and T3 were included. At the end of recovery time (T5), 72 skin mucosa samples (1-time × 3 regimes × 8 tanks × 3 replicates) and 72 gut samples (1-time × 3 regimes × 8 tanks × 3 replicates) were collected from the host. In addition, 240 samples (5 times × 3 regimes × 8 tanks × 2 technical replicates) of water (2 L) microbial filter (0.22 μm) were sampled between T3 and T5 at an interval time of 15 days corresponding to five recovery time points (TR1, TR2, TR3, TR4, and T5).

### Metal concentration in water and fish liver

Every week until the end of the CdCl_2_ exposure regimes, we measured the concentration of trace metals of cadmium (Cd), zinc (Zn), and copper (Cu) in the yellow perch liver and water tanks using the ICPMS (Ionization coupled mass spectrometry) technology available at INRS (Institut National de la Recherche Scientifique). For further details of the measurement of Cd in liver preceded by acid digestion and lyophilization see our under review study Cheaib et al. (2019). Two-way analysis of variance (ANOVA), Tukey’s test, and Wilcoxon rank test were applied to test the significance of cadmium accumulation in liver and water over time, and between treatment groups.

### DNA extraction, libraries preparation, and 16S amplicons sequencing

DNA was extracted using the Qiagen DNeasy blood and tissue kit for skin mucosa, and TRIzol organic phase followed by BEB (back extraction buffer) and PCI (phenol/chloroform/isoamyl alcohol 25:24:1) solutions for all gut samples. The V3–V4 hypervariable region of the universal rDNA 16S gene (Werner et al. 2012) was amplified using universal specific primers. The libraries of amplicons were prepared using a set of 384 combinations of adaptors, processed in one sequencing run, on an Illumina Miseq sequencing machine. Reactions of PCR were verified by electrophoresis on 2% agarose gel, purified, and quantified by fluorescence for the double-strand DNA concentration using Quant-iT™ PicoGreen™ dsDNA Assay Kit (Thermo Fischer Scientific).

### Bioinformatics and biostatistics analyses

#### Reads preprocessing and OTUs clustering

Sequence analysis was performed with our bioinformatic pipeline as described previously [42, 43]. In the first instance, we used SICKLE Version 1.2 to trim the reads (>Q30 Phred quality score) followed by utilizing PANDASEQ Version 2.11 [44] assembler for merging paired-end read into a single merged reads (~ 350 bp) corresponding to the amplified 16S rRNA V3–V4 hypervariable region (347 F-805 R). Based on an approach of de novo sequence clustering before the taxonomic assignment, reads were clustered into OTUs at 97% identity with USEARCH Version 9 (Edgar RC. 2010) and filtered out using UNOISE2 algorithm [45] to discard chimeric sequences, putatively produced during PCR amplification cycles using OTUs were annotated using RDP database as previously described in our pipeline [42, 43] to. Community structure and composition of metacommunities were analyzed across time and treatments by richness (OTUs count), evenness (Shannon index), and the Gunifrac phylogenetic distance [46] using the vegan [47] and Rhea [48] packages in R.

We then calculated, the alpha-diversity indices (richness and evenness) and beta-diversity (phylogenetic distance) differences between experimental groups and used rank statistics tests (Kruskal-Wallis/Wilcoxon) to assess their significance. The resulting *p* values for pairwise comparisons in alpha and beta-diversity were corrected for multiple testing using the Benjamini-Hochberg method (Benjamini and Hochberg, 1995). Note that the Beta-diversity was calculated using the generalized UniFrac metric [49], which considers both the dominant and the rare OTUs. The permutational multivariate analysis of variance (henceforth referred to as PERMANOVA) was applied to the Gunifrac distance matrices to explain the sources of variations including the experimental groups. To test homogeneity of variances, we performed the multivariate homogeneity test which a Multi-Response Permutation Procedure (MRRP) of within versus among group dissimilarities dispersions of Gunifrac distances. The non-metric multi-dimensional scaling (NMDS) was performed to visualize Gunifrac distances in a reduced space with *k* = 2 dimensions. For statistics comparison of one-dimensional statistics of multiple groups, we used the non-parametric Kruskal-Wallis rank-sum test because of the strong assumption of the normal distribution of OTUs abundance being rarely assumed.

The alpha-diversity variation across time and per treatment was predicted and plotted with linear mixed effect models using the ratio of richness/evenness as a response variable, time, and cadmium concentrations in water and liver as fixed effects, with the categorical variable tank as a random effect.

Using the lmer R package, for water, the model was used as following in R:

Model<- lmer (Richness/Shannon.effective~Time+Cd.Water+(1|Tank), data=mixdata, REML = TRUE)

whereas for each host habitat (Skin, Gut), we employed the following model:

Model_host<- lmer(Richness/Shannon.effective~Time+Cd.Liver+Cd.Water+(1|Tank), data=mixdata, REML = TRUE)

The confidence interval was then predicted using the predict interval() function in R.

### Post-OTUs analysis, networks, and function prediction

Structure and diversity measures of groups (control and treatments) were compared with rank statistics tests (Kruskal-Wallis/Wilcoxon) adjusted with BH (Benjamin-Hochberg) test for multiple corrections, and the *p* value < 0.05 as a threshold of statistical significance. To understand the role of relative abundance of OTUs on the similarities of community structure, correlation networks of communities (samples) were constructed using the Spearman coefficient as a robust approach of correlation detection [50]. Significant positive and negative correlations were filtered and false discovery rate (FDR) was assessed with B-H test for multiple corrections. Next, network visualization and analysis were performed with Cytoscape software [51]. The network centrality was analyzed using the “Network Analyzer” plugin in Cytoscape. The betweenness centrality of a node was calculated as the total number of the shortest paths from all nodes to all other nodes that pass this node [52]. The centrality of the nodes reflected their importance in transmitting information between hubs; it does not depend on the feature of node degree, which describes the total node connectivity. The size of nodes was proportional to the number of OTUs in each sample, and the coefficient of the significant correlation between two nodes was inversely proportional to the size of the edge. Finally, functional profiles of each community type at every time point were predicted using the software TaxforFun [53].

### Neutral and deterministic models to asses the recovery of community assembly

In the null hypothesis, the neutral model [16] (Sloan et al. 2006) assumes a beta distribution of OTU abundance. Using the non-linear partial least square method [41], which estimates the migration rate (m) of OTUs from their source to a destination community, the model predicts the frequencies of OTUs. The estimated migration rate (m) is the probability that a random loss (death or emigration) of an OTU in a destination community is replaced by dispersal from the source community. Comparing the predicted versus observed frequencies, we can determine which OTU fits the model in each host and water community, at every time point, across both control and treatment groups. The goodness of fit to the model was measured using the coefficient of determination *R*^2^ (*R*^2^ > 0.5) within a confidence interval of 95%, where increased strength of goodness of fit to the model suggests an essential role of stochastic processes in the microbiome assembly.

## Results

### Cadmium concentration bioaccumulation in the fish liver during recovery time

Interestingly, the concentration of cadmium ions measured with ICPMS increased significantly in the fish liver even after 2 months from stopping exposure. The Cd concentration increased from 0.4 ppb to 1 ppb in the variable CdCl_2_ regime (CV) and from 0.5 ppb to 1.17 ppb in the constant CdCl_2_ regime (CC). However, in the water, as expected, the Cd concentration significantly decreased from 6.4 ppb to 1.06 ppb in CV, and from 5.8 ppb to 1.34 ppb in CC (Tables 1 and 2). Consequently, the accumulation of Cd in liver and water was always significantly higher in treatments CC and CV compared to the control group (Table 3). Similar Cd concentrations observed among treatment groups CC and CV in the water at times T3 and T5 (expected at maximum Cd concentration added in tanks) were observed in fish liver only at time T5 (Table 3).

**Table 1:** Statistics of Cd concentrations in water and fish liver over time and treatments

	Liver			Water		
Cadmium average concentration (ng/ml)	T0	T3	T5	T0	T3	T5
Ctrl	0.0860	0.1578	0.2330	0.0650	0.0750	0.0388 *
CV	0.0860	0.4000	1.0015	0.0670	6.4300	1.0600
CC	0.0860	0.5235	1.1700	0.0980	5.8000	1.3400

*p* value < = 0.05 : “*”

**Table 2:** Statistics of cadmium concentration variation over time in water tanks and fish livers

	Liver			Water		
Overtime comparisons	T0–T3	T0–t5	T3–t5	T0–t3	T0–t5	T3–t5
Groups	Tukey’s *p* value			Wilcox’s *p* value		
Ctrl-Ctrl	1.0000	0.8253	0.8112	1.0000	1.0000	1.0000
Cv-CV	0.0003 ***	0.0000 ***	0.0000 ***	0.0000 ***	0.0000 ***	0.0000 ***
CC-CC	0.0000 ***	0.0000 ***	0.0000 ***	0.0000 ***	0.0000 ***	0.0000 ***

*p* value < = 0.001 : “***”

**Table 3:** Statistics of cadmium concentration variation among treatments in water tanks and fish livers

	T3		T5	
Statistics tests (Tukey, Wilcox)	Liver	Water	Liver	Water
Groups	Tukey’s *p* value	Wilcox’s *p* value	Tukey’s *p* value	Wilcox’s *p* value
Ctrl-CV	0.00002 ***	0 ***	0.0000 ***	0.0002 ***
Ctrl-CC	0.0009 ***	0 ***	0.0000 ***	0.0002 ***
CC-CV	0.0281 *	0.1304	0.5362	0.1304
All (Kruskal−Wallis rank-sum test)	0.0002 ***	0 ***	0.0000 ***	0.0003 ***

*p* value < = 0.05 : “*”

*p* value < = 0.001 : “***”

### Genotypic signatures of community recovery

At the alpha-diversity level, to investigate how far diversity metrics could be used as indicators for metacommunity structure recovery, both richness and the evenness were calculated in water and host-microbial communities. In the host microbiome, time had a significant effect on diversity measures within all groups between times T3 and T5. The richness and evenness have significantly increased over time in skin microbiota and significantly decreased in the gut microbiota. Over the five recovery time points (TR1, TR2, TR3, TR4, T5), temporal comparisons in the water microbial communities associated with each experimental group did not show a significant change of evenness for CC and CV, but did during TR2–TR4 within the control group (Ctrl). Within these communities, a significant change in richness during the whole recovery period except TR2–TR3 was also found for CC and CV (Additional file 7: Table S1a-b).

In contrast, both richness and evenness in the control group of skin microbiota significantly fluctuated over the recovery period (T3–T5, after CdCl_2_ addition had stopped). At time T3, the pairwise comparison of CC and CV against the control group (CC-Ctrl and CV-Ctrl) revealed significant differences in microbial richness in the gut and evenness in the skin. At time T5, statistical tests did not detect any significant change in diversity measures between all groups for gut and skin microbiome (Additional file 7: Table S1-c); however, as at T3, the evenness of skin microbiome at T5 was significantly divergent between cadmium treatments, (*p* value = 0.0063) (Additional file 7: Table S1-c).

The comparative analysis of richness and evenness among water and host microbiota showed convergent patterns of diversity between the water and the skin communities before the disturbance and after the recovery (Additional file 1: Figure S1).

**Fig. 2:**
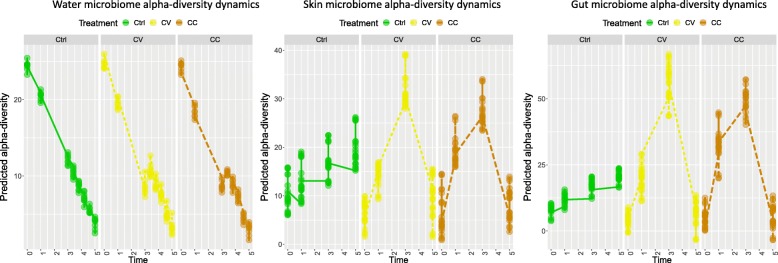
Predicted alpha-diversity plots by linear mixed model. Alpha-diversity in water and host-microbial communities over time and among treatments is predicted using the linear mixed model. The richness/evenness ratio were considered as response variables, the fixed effects were defined by time and cadmium concentration (in water and liver), and tanks were taken as random effects. Over time, the predicted alpha diversity in host microbial communities (skin, gut) highlights stable trends of the Control group compared to the treatments. However, all groups of the water microbial communities decrease overtime. Constant cadmium regime (CC) is in orange, variable cadmium regime (CV) is in yellow, and control (control) is in green

The predicted alpha-diversity values along with the fitted linear mixed effect model for water communities showed a significant drop in treatment values compared to the control group. On the other hand, for the host communities (skin and gut), they increased under the selection regimes and decreased during the recovery time span (Fig. 2).

In summary, except for the linear mixed effect model results, the observed patterns of alpha diversity metrics changes across the experiment did not show a clear trend over the course of the experiment. Nonetheless, increasing evenness and richness was a general trend for the skin while decreasing evenness and increasing richness was representative of the gut microbiome community recovery.

Beta diversity (Gunifrac) between samples was compared using a PERMANOVA and a multivariate test for variance homoscedasticity. By T3—at peak cadmium exposure—significant differences (*p* < 0.05) among treatments were observed in all microbial communities of water and host (Table 4 ; Additional file 2: Figure S2); and by T5, both variable (CV) and constant (CC) cadmium exposure treatments retained differences in skin communities compared to the controls despite the recovery period. Surprisingly, given our expectation that cadmium exposure would have a major impact on community recovery, a high similarity in the community phylogenetic structure between the control and CC groups was detected among gut microbial communities at T5. Beta-diversity between treatments (CC, CV, Ctrl) was always significantly divergent at each time point in water samples except for the observed convergence between CC and CV at recovery time TR2 (Table 4; Additional file 2: Figure S2). The comparison of beta-diversity showed a community structure divergence (*p* value < 0.001) between water, skin, and the gut microbiota before the disturbance and after the recovery (Additional file 3: Figure S3). The results show that water microbiome at time T3 is not representative enough of fish microbiome (see the blue cluster in the phylogram of CC, page 2 of Additional file 3: Figure S3). However, at the recovery time T5, the water was not representative of the fish microbiome in gradual selection regime (see the blue cluster in the phylogram of CV, page 3 of Additional file 3: Figure S3).

**Table 4:** Phylogenetic divergence in host and water microbiomes

	Time	Groups	Permanova	Multiple test correction	Betadisper	Mrpp
			*p* value ADONIS	*p* values B-H	*p* values dispersion	*p* value MRPP
Ctrl = control regime; CC = concentration is constant; CV = concentration is variable						
Gut	T0	All groups	0.052		0.079	0.279
		CC-Ctrl	0.042 *	0.077	0.048*	0.033 *
		CC-CV	0.051	0.077	0.141	0.049 *
		Ctrl-CV	0.280*	0.280	0.302	0.234
	T3	All groups	0.001**		0.367	0.647
		CC-Ctrl	0.001**	0.002 **	0.916	0.001***
		CC-CV	0.006 **	0.006 **	0.145	0.008 *
		Ctrl-CV	0.001***	0.002 **	0.295	0.001 ***
	T5	All groups	0.006 *		0.217	0.199
		CC-Ctrl	0.135	0.135	0.923	0.104
		CC-CV	0.005 **	0.012 *	0.084	0.009**
		Ctrl-CV	0.008 **	0.012 *	0.130	0.008 **
Skin	T0	All groups	0.016 *		0.540	0.500
		CC-Ctrl	0.166	0.166	0.649	0.154
		CC-CV	0.081	0.122	0.256	0.075
		Ctrl-CV	0.026 *	0.078	0.599	0.020 *
	T3	All groups	0.008 **		0.820	0.580
		CC-Ctrl	0.035*	0.049 *	0.619	0.049 *
		CC-CV	0.049 *	0.049 *	0.586	0.045 *
		Ctrl-CV	0.021*	0.049 *	0.872	0.009 **
	T5	All groups	0.001***		0.380	0.001 ***
		CC-Ctrl	0.011 *	0.011 *	0.749	0.008 **
		CC-CV	0.001***	0.002 **	0.307	0.002 **
		Ctrl-CV	0.001 ***	0.002 **	0.236	0.001 **
Water	T0	All groups	0.001 ***		0.656	0.886
		CC-Ctrl	0.018 *	0.027 *	1.000	0.009 **
		CC-CV	0.002 **	0.006 **	0.291	0.002 **
		Ctrl-CV	0.036 *	0.036 *	0.519	0.038 *
	T3	All groups	0.001 ***		0.036 *	0.596
		CC-Ctrl	0.002 **	0.002**	0.068	0.002 **
		CC-CV	0.001 **	0.002 **	0.020 *	0.001 ***
		Ctrl-CV	0.001 ***	0.002 **	0.546	0.001 ***
	TR1	All groups	0.001 ***		0.214	0.108
		CC-Ctrl	0.001 ***	0.002 **	0.305	0.002 **
		CC-CV	0.001 ***	0.002 **	0.064	0.001 ***
		Ctrl-CV	0.007 **	0.007 **	0.653	0.007 **
	TR2	All groups	0.001 ***		0.639	0.381
		CC-Ctrl	0.003 **	0.005 **	0.395	0.003 **
		CC-CV	0.096	0.096	0.892	0.077
		Ctrl-CV	0.001 ***	0.003 **	0.391	0.001 ***
	TR3	All groups	0.001 ***		0.561	0.629
		CC-Ctrl	0.001 ***	0.003 **	0.343	0.001 ***
		CC-CV	0.003 **	0.005 **	0.536	0.002 **
		Ctrl-CV	0.007 **	0.007 **	0.620	0.006 **
	TR4	All groups	0.001 ***		0.498	0.031 *
		CC-Ctrl	0.009 **	0.009 **	0.536	0.007 **
		CC-CV	0.006 **	0.009 **	0.179	0.009 **
		Ctrl-CV	0.004 **	0.009 **	0.578	0.002 **
	T5	All groups	0.001 ***		0.408	0.027 *
		CC-Ctrl	0.002 **	0.002 **	0.331	0.001 **
		CC-CV	0.001 ***	0.002 **	0.578	0.003 **
		Ctrl-CV	0.002 **	0.002 **	0.438	0.001 **

The phylogenetic distances between OTUs were computed using Gunifrac distance (see “Methods” section). The divergence between treatments and control was assessed using PERMANOVA and the homogeneity for group dispersions (distance from centroid) was evaluated using two multivariate tests, BETADISPER and multi-response permutation procedure (MRRP) of within versus among group dissimilarities. The significance of divergence between groups was measured by applying multiple correction tests with Benjamini-Hochberg BH (*p* value < 0.05)

*p* value < = 0.001 : “***”, *p* value < = 0.01 : “**”, *p* value < = 0.05 : “*”

### Microbial taxonomic composition change during recovery

**Fig. 3:**
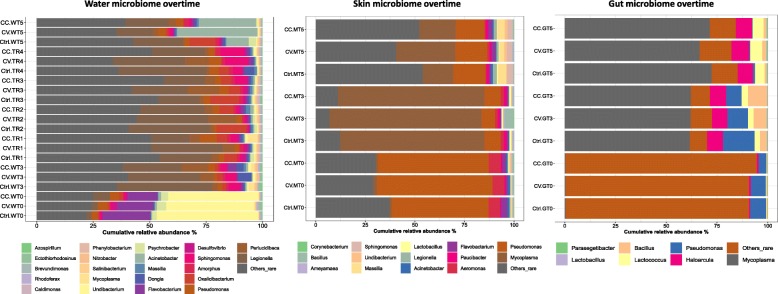
Taxonomic composition dynamics of host communities. Stacked barplots show the most abundant taxa (> 0.5%) overtime in the gut, skin and water microbiomes. The genera that significantly changed among treatments and control at T5 are summarized in Additional file 8: Table S2

At T5, no significant changes were observed between groups at the phyla level in the water, but Actinobacteria in the gut, and both Euryarchaeota and Tenericutes in the skin, were significantly different between control and treatments (CC and CV). Additional file 8: Table S2 details several taxa that showed significant differential abundance between treatments (Ctrl, CV, CC). Of particular importance, putative pathogenic genus *Flavobacterium* was significantly enriched in the skin for both groups of CdCl_2_ exposed fish at T5, despite the recovery period. In the gut microbiome, *Syntrophococcus* was the only genus to be significantly different between treatments (Fig. 3; Additional file 8: Table S2). In the water, significant differences in taxonomic abundance between CV and Ctrl were restricted to one genus (*Kiloniella*) at recovery time TR1 and two genera (*Marinobacter* and *Perlucidibaca*) at T5. No significant differences in taxonomic composition were detected between CC and Ctrl in the water at T5. Overall, statistical analysis of taxonomic composition dynamics over time within each treatment during the recovery period revealed several minor differences (see Additional file 9: Tables S3 for more details).

**Fig. 4:**
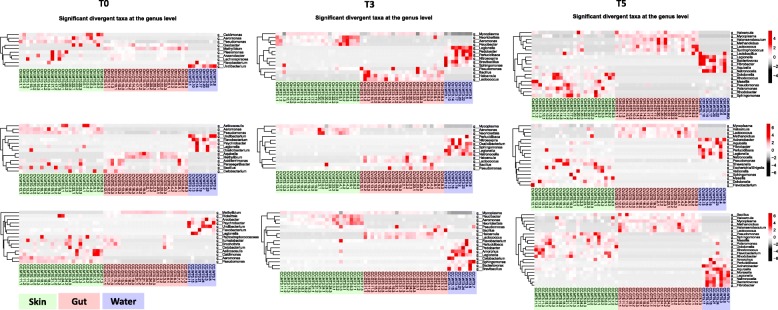
Heatmaps of differential abundance among host and water communities. This figure from left to right includes 9 heatmaps of the significant taxonomic fingerprints at the genus level between gut, the skin and the water at times T0 (first column), T3 (second column), and T5 (third column) in the control (first row), the CV (second row), and the CC (third row) groups. The hierarchical clustering of the relative abundance of phyla which significantly changed over time was performed using Ward’s method and Bray–Curtis dissimilarity distance. Vegan package and pheatmap () function in R were used

On the other hand, the pairwise comparison of taxonomic composition between different type of communities at each time point and for each experimental group showed significant divergence between microbial communities of gut, skin, and water. At the recovery time (T5), *Tenericutes*, *Euryarchaeota*, and *Firmicutes* were inherently associated (significantly upregulated) with the gut microbiome; *Actinobacteria* and *Bacteroidetes*, on the other hand, were specific to skin microbiome; with *Fibrobacteres* and *Actinobacteria* implicated with the water microbiome. Despite all this, the *Proteobacteria* were found to be prevalent and common in water and skin microbiome. At the selection time (T3), *Fibrobacteria* and *Actinobacteria* were scarcely abundant and were picked up as differentially abundant (Additional file 6: Figure S6). To delineate the most relevant taxa (at the genus level) significantly changing between the communities, we have performed a pairwise test on an overall comparison of skin, gut, and water at each time point for different treatment groups. The results plotted in heatmaps in the Fig. 4 clearly reveal that each community type has an inherent signature and the corresponding proportions of genera differed between control and treatments over time with high similarity between the CC and Ctrl at time T5 (Fig. 4; Additional file 10: Table S4).

Correlational analysis (Additional file 4: Figure S4) revealed a positive relationship between specific genera and concentrations of cadmium in perch liver and water. In aquaria treated with CdCl_2_ (CV and CC), cadmium concentrations in water and liver showed strong significant positive correlations with seven genera from the gut microbiome, each of which represented a different phylum and had a strong negative correlation with the relative abundance of *Mycoplasma*. In the skin microbiome of both CC and CV, 15 genera (*Sphingomonas; Haloarcula; Legionella; Flavobacterium; Ameyamaea; Dokdonella; Shigella; Massilia; Mycoplasma; Polaromonas; Pseudomonas; Rhodobacter; Rhodococcus; Shewanella*; *Syntrophococcus*) showed significant profiles of positive or negative correlations with the Cd concentrations in the liver (Additional file 4: Figure S4). Divergent profiles between CC and CV were only observed for the correlations of *Shewanella* and *Syntrophococcus* with cadmium concentrations. Similar correlation profiles between these groups were observed in the water (Additional file 5: Figure S5).

### Correlational networks of host and water microbiome

**Fig. 5:**
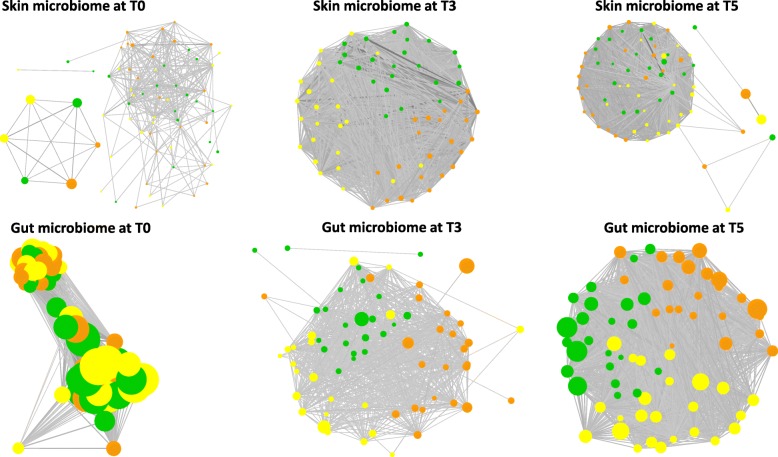
Recovery dynamics of the networks of host communities. The networks organization is based on nodes betweenness centrality among treatments and Control. Unstructured patterns in the networks were observed at T0. Node size represents sample richness. The strength of correlation (Spearman correlation from 0.3 to 1) between two nodes is inversely proportional to the size of the edge. This network was built using R and Cytoscape software. Constant Cadmium samples (CC) are in orange, variable cadmium samples (CV) are in yellow, and control (Control) samples are in green

**Fig. 6:**
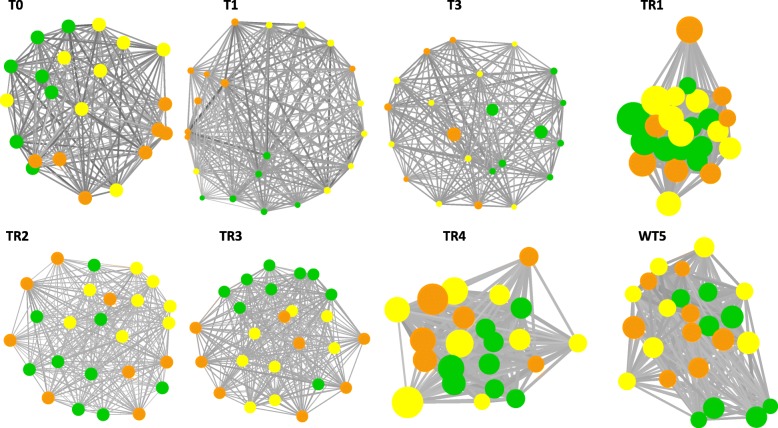
Recovery dynamics of the network of water communities. The networks organization at every resilience time TR1, TR2, TR3, TR4, and WT5 is based on nodes betweenness centrality among treatments and control. The network modules easliy distinguishable between groups since T0. The size of nodes (sample richness) at the beginning of TR1 and at the end of the time TR4 showed shifts in the community richness. The strength of correlation (Corr. Spearman from 0.5 to 1) between two nodes is inversely proportional to the size of the edge. This network was built using R and Cytoscape software. constant cadmium samples (CC) are in orange, variable cadmium samples (CV) are in yellow, and control (control) samples are in green

**Fig. 7:**
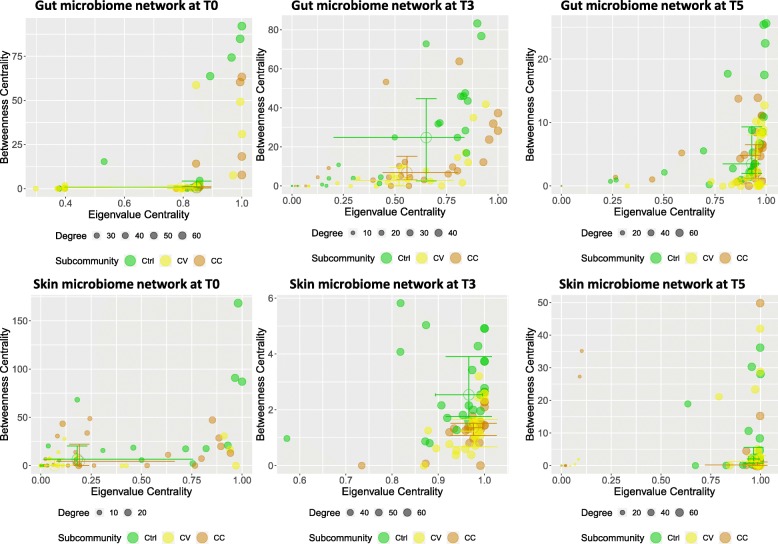
Centrality plots of host microbiome networks. This figure summarizes relationships of betweenness centrality versus eigenvalue centrality of host microbiome networks among treatments and at each time point. The results show evidence of shift in centrality medians between the control regime (which is higher) and the cadmium selection regimes. The plots of eigenvalue centrality versus betweenness centrality clearly reveal that centrality shift at time T3 for skin and gut microbiome

In the host and water communities, the network analysis of samples correlations showed a partitioned community distribution between treatment groups, at time T3, and overlapping patterns during the recovery period (Figs. 5 and 6). At time T0, the correlational networks of host microbiome showed unstructured topology with on average a fewer number of edges. An edge can represent significant low correlations (Spearman’s Rho correlation > 0.5) between samples from different groups with the node size proportional to the richness of each sample. The topological distribution of nodes in the network was further analyzed by comparing the betweenness centrality to the eigenvalue centrality (Fig. 7). The results indicate a shift in the mean of the centrality metrics between the control (which is higher) and the cadmium selection regimes. The plots of eigenvalue centrality versus betweenness centrality clearly reveal that these communities shift at times T3 for skin microbiome, as well as at time T3 for gut microbiome. The high betweenness centrality observed in control reflects the efficiency of network centrality measure to predict the effect of perturbation on the community structure during the selection phase, but not during the recovery time as centrality median shifts at T5 was not observed (Fig. 7). The same centrality analysis was obtained for water microbiome networks resulting in similar patterns at time T3 (results not shown).

### Recovery of microbial functional diversity a time T5

**Fig. 8:**
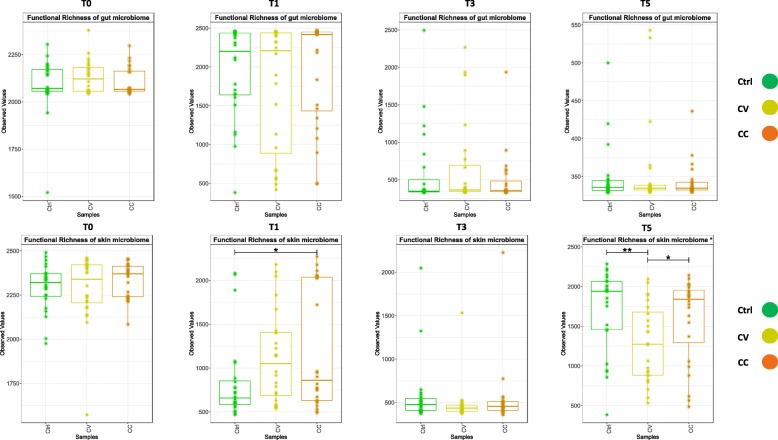
Function diversity dynamics in host and water microbiome. Boxplots of functions profiles were predicted from the matrices of taxa count using the software Tax4Fun. The statistical significance (*p* value < 0.05) found using ANOVA followed by FDR (false discovery rate) test are represented with asterisks points (0.001: “***,” 0.01: “**,” 0.05: “*”)

At time T0, an ANOVA of functional richness within the metacommunity showed a significantly higher average of functional diversity in gut and skin microbiomes compared to water microbial communities. Surprisingly stable in water communities, functional diversity did not show any significant divergence among the treatment and control group at T3 regardless of the community type (skin, water, gut). The lack of treatment effect observed may well have been masked by the strong influence of time over microbial diversity (Cheaib et al. 2019 submitted ISMEJ). However, at T5, the functional diversity of skin microbiome was significantly higher in the control group than in treatment groups according to the ANOVA (CC-CV_(*p* value)_ = 0.04; CV-Ctrl_(*p* value)_ = 0.0055; CC-Ctrl_(*p* value)_ = 0.45) (Fig. 8). In the gut microbiota, no significant changes in functional diversity were detected between treatments (CC-CV_(*p* value)_ = 0.3; CV-Ctrl _(*p* value)_ = 0.54; CC-Ctrl _(*p* value)_ = 0.58).

### The role of neutral and deterministic processes in the recovery of host microbiota

**Fig. 9:**
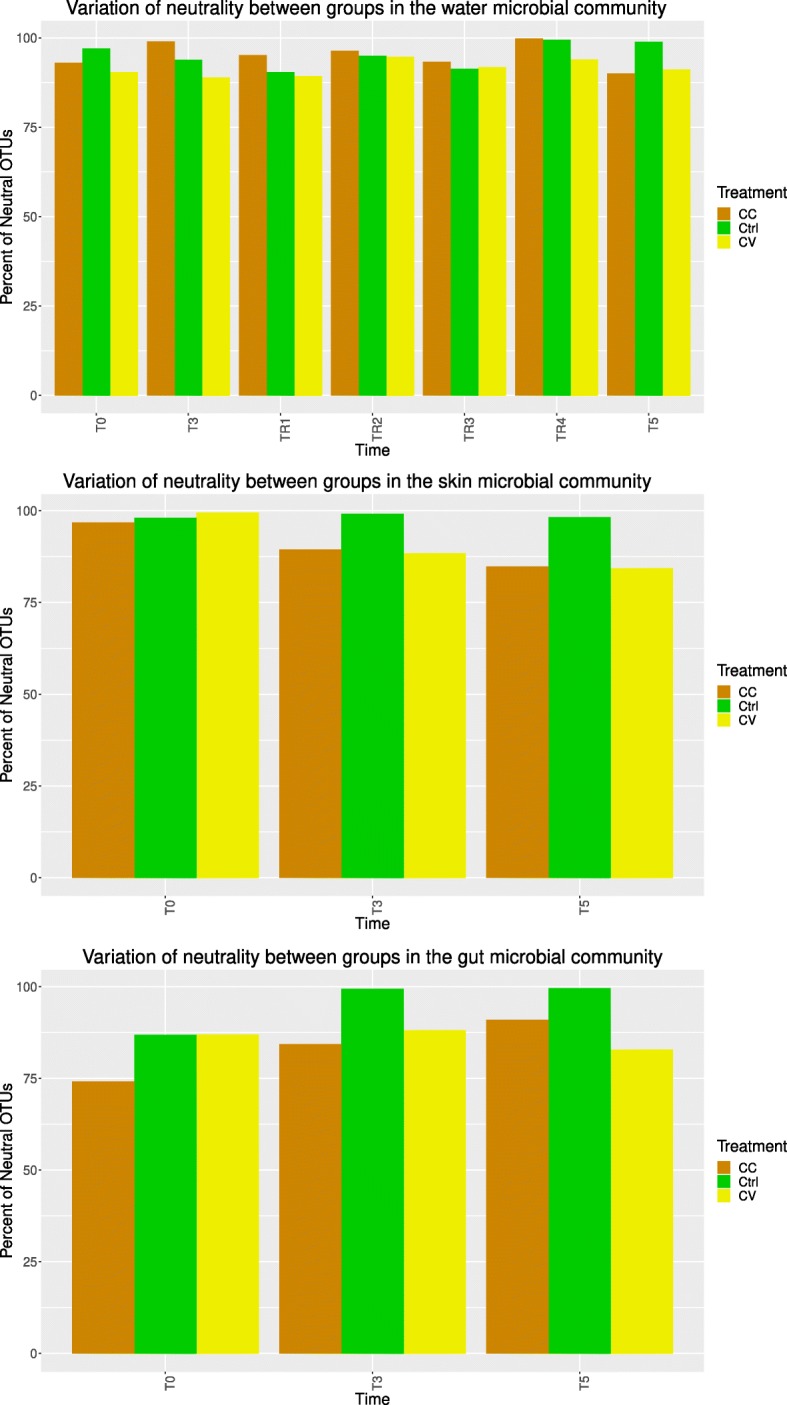
Percentage of neutral OTUs over time and treatment. Using the non-linear least squares model (NLS), the percentage of OTUs that fit the neutral model within a confidence interval of 95% showed variable trends between communities across time and treatments. A goodness of fit *R*^2^> 0.5 was considered as the significant threshold of neutrality fit. The cadmium treatment invoked stochasticity in the water communities, while in gut and skin communities, the percentage of neutral OTUs remained higher in the control compared to treatments.

**Fig. 10:**
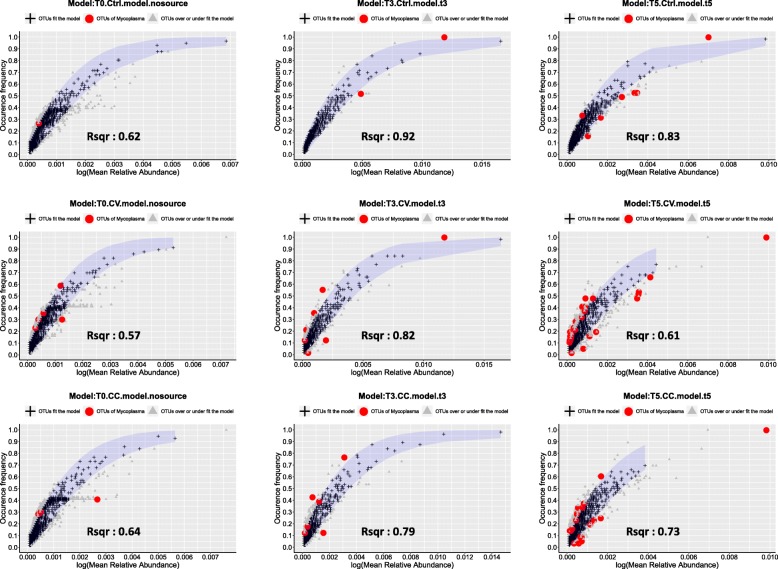
Demographic variation of metacommunity neutrality across water and host microbiome. This figure summarizes the scatterplots of neutral model fitting the whole metacommunity ( gut skin and water) at times T0 (first column), T3 (second column) and T5 (third column) in the control (first row), the CV (second row), and the CC (third row) groups. Neutral OTUs are shown in black, non-neutral are depicted in grey, while the red is *Mycoplasma* sp. OTUs. We see no *Mycoplasma* sp. OTUs that fit the neutral model in the whole metacommunity

The goodness of fit of host and water microbial communities to the non-linear partial least square model (NLS) was high (*R*^2^ > 0.5), supporting the theory of predominant neutrality (Additional file 11: Table S5). To disentangle gut and skin microbiota ontogeny from the cadmium effect, the NLS model was deployed using the control as a reference. A comparison of observed versus predicted OTU frequencies revealed that the percentage of neutral OTUs in skin and gut microbiota (Fig. 9) at the recovery time T5 is higher in the control group compared to those in treatments at T3 and T5. The same analysis was undertaken in the water and the percentage of neutral versus non-neutral OTUs showed the same trends across Ctrl, CC, and CV at T5. Overall, we noted a preponderance of OTUs that fitted the neutral model in all comparisons. The majority of the OTUs that did not fit the neutral model was assigned to Mycoplasma species (indeed no mycoplasma sp. OTUs fitted the neutral model), which can be seen in Fig. 10 as well as Additional file 12: Table S6 and Additional file 13: Table S7. The neutral process was much more prevalent in the control group at time T3 and T5 as compared to the treatment groups.

## Discussion

Our data clearly show that long-term bioaccumulation of cadmium occurs in the *Perca flavescens* liver on exposure to aqueous cadmium salts. Our data also showed that the cadmium persists at high concentrations even once the treatment has been stopped for 2 months. We have already shown that cadmium treatment clearly impacts both the skin and gut microbial communities, as compared to controls (Cheaib et al. 2019, under review). Recovery was the focus of the current study, and microbial communities post-exposure showed different routes to (and extents of) recovery in those associated with the skin and gut once cadmium treatment was ceased. In the skin, evenness—the extent to which different microbes in a community share similar abundances—and richness increased during the recovery phase in cadmium-treated fish. Beta-diversity comparisons, meanwhile, revealed significant differences between all experimental cohorts (Ctrl, CC, CV) in water and skin niches. Among gut microbial communities, decreasing richness and increasing evenness were observed over the recovery period. Beta-diversity metrics indicated few significant differences between cadmium and control treatments. Crucially, by the end of the recovery period in the gut, functional richness was comparable between tests and control, a potential signal of full community recovery. We used models to assess the relative roles of microbial assembly in the different groups. We found evidence that neutral processes were a more prevalent contributor to microbial community turnover in control treatments than in those treated with CdCl_2_—likely indicating the role of selective processes in driving community recovery. Overall our data do not strongly support our prediction that the most extreme cadmium exposure (CC) would lead to the least successful recovery. Instead, CC and CV treatments, especially in the gut, demonstrated a good degree of recovery, both in terms of both alpha and functional diversity.

At the end of the third month of exposure (T3), the cadmium concentration in the liver was significantly higher in CC and CV than in the control group. These concentration differences were still observed two months (T5) after the gradual clearing of Cd started. The liver plays a major role in the accumulation, excretion, and biotransformation of contaminants like metalloids [54, 55], and bioaccumulated metals remain at high concentrations in the liver due to its depuration function of other organs (such as gills and muscles) [56]. Long-term bioaccumulation of cadmium has been documented in perch and other biological systems [39, 57–60], as has its effect on ecosystem services in soil and water [34, 61] and metazoan gut ecosystems [62–65]. This study not only confirms the chronic bioaccumulative effect of Cd but also suggests that the sequestered Cd in perch liver presumably cannot predict the regime of exposure (CC, CV), as the concentration did not significantly vary in livers between both regimes, CC and CV, at T5. Over the recovery period, the concentration of Cd in water significantly decreased, but Cd was not completely removed from the tank system since it has a strong affinity to the tank silicone gaskets, and has high competitiveness with Zn for the debris of organic compounds always available in the water aquarium ecosystem [66].

The water microbial communities showed few differential abundances of taxa differences during the recovery period (Fig. 3). Furthermore, the microbial functional diversity in water remained stable throughout the experiment, and no significant differences between treatments were found during the exposure or the recovery periods. However, the community beta-diversity at the phylogenetical level between treatments (CC, CV, Ctrl) showed significant difference at each time point, suggesting a pattern of taxon-function decoupling as an adaptive strategy reported previously in lacustrine water contaminated with cadmium [34].

To assess the yellow perch microbiome recovery, we examined alpha-diversity (richness and evenness), beta-diversity (phylogenetic distance), taxonomic composition, and functional diversity (metabolic functions). Most of these measurements are commonly used as community-wide metrics to assess the recovery of microbial communities, for example, in humans [12, 67], soil [22, 68], and wastewater [69] (Vrieze et al. 2017).

In the skin microbiome, the disturbance intensity (cadmium gradient) had a differential impact on the community recovery trajectories, resulting in a significant difference of evenness (Additional file 7: Table S1c) and functional diversity (Fig. 8) between CC and CV at time T5 (Table 4). During the gradual exposure regime (CV), the cadmium may provoke an endurance effect on the skin microbiota which was progressively adapted to the cadmium accumulating in the tank system, while within the constant exposure regime (CC), abrupt diversity and taxonomic changes might have been triggered. Gradual changes are evident under stress gradients, for example, within bioreactors, the anaerobic microbiome has been shown to gradually adapt following ammonium disturbanc e[70]. Consequently, the significant divergence in the functional diversity between CV-Ctrl and CV-CC, not between CC-Ctrl, perhaps indicates a unique adaptive evolution signature of skin microbiome under CV regime. Therefore, the skin communities from CV and CC may have followed a different recovery trajectory after adaptation. Strikingly, the recovery of skin microbiota of the most extreme exposure (CC) appeared to be the most successful, when considering the convergence of richness, evenness, and functional diversity between CC and Ctrl. However, significant differences among CC, CV, and Ctrl in terms of phylogenetic divergence (Table 4) and taxonomic composition shifts (Fig. 3, Additional file 8: Table S2; Additional file 9: Table S3; Additional file 10: Table S4) suggest this recovery was incomplete. For instance, a significant increase in fish pathogens like *Flavobacterium*, *Legionella* and opportunists like *Mycoplasma* was detected in both cadmium groups (CC and CV) compared to control. The relative abundance of *Flavobacterium* was significantly lower in the control group with a low percentage (< 0.5 %). Perturbation with cadmium can facilitate the proliferation of opportunistic pathogens, this concern has been found in other studies of fish microbiota recovery after exposure to antibiotic [71] and triclosan biocide [72]. Similar taxonomic changes in both exposure regimes (CC and CV) were expected [73]. Overall, the cadmium disturbance may cause a shift to an alternative stable state, demonstrating differential and incomplete recovery of the skin microbiota in CC and CV.

In the gut microbiome, the recovery routes were different; at time T5, there was only a significant evenness convergence between CC and CV. Overall, the few significant differences in taxonomy, as well as the phylogenetic divergence (Additional file 8: Table S2) between CC-CV and Ctrl-CV, but not between CC-Ctrl, suggests a full recovery of the gut microbiota in CC and gradual recovery in CV. At the level of taxonomic composition, overall the dominance of opportunists *Tenericutes* was also a feature of farmed Eurasian perch *(Perca fluviatilis*) gut microbiota studied in a context of stress predation [74]*,* although they were not found in the wild Eurasian perch [75].

In the skin and gut microbiota, the significant increase in diversity (evenness and richness) over the recovery period (T3–T5) was consistent with the diversity increase in other host-associated studies such as the recovery of the fathead minnow gut microbiome from a low-level triclosan exposure [72], the human intestinal microbiota post-infection [67], the murine gut microbiome exposed to antibiotics in early life [76], and the molasses wastewater [69]. Further, the functional redundancy observed in all water and gut microbial communities is a major adaptive strategy behind resistance and recovery [34, 69]. Lastly, the significant divergence of skin and gut microbiota diversity over time within the control group suggests persistent divergence from the initial community structure due to microbiota ontogeny through the developmental stage of fish juveniles [41].

Our findings demonstrate a relative role of neutral processes shaping the bacterial communities’ recovery following exposition to metallic stressors. According to the neutral model fit, the percentage of neutral OTUs in skin and gut microbiota was significantly higher in the control group compared to CdCl_2_ treated groups, which provides evidence that neutral processes are the major contributor in the microbiota assembly in non-stressed yellow perch, therefore suggesting that selective processes are at play in driving the community recovery in stress-exposed groups. Furthermore, Mycoplasma sp. are a dominant species in perch microbiome, implicated in literature for other fish species [43, 77]. The inability of neutral models to explain the abundance of any OTUs for Mycoplasma sp. in the current study suggests that these bacterial strains can quickly adapt to the host environment. Our study is the first to investigate the relative importance of neutrality and determinism in driving post-disturbance assembly of the host-associated microbiome.

## Conclusions

This study not only elucidates the long-term bioaccumulation effect of toxic metals on biological systems but also suggests that the sequestered cadmium in the fish liver will not likely predict the magnitude of exposure regime (constant or variable). The effect of cadmium exposure on microbial communities is also varying and dependent on the nature of the host it is originating from. Surprisingly, after recovery, skin and gut microbiota of fish exposed to constant concentrations of cadmium (CC) were closer to the control group than those exposed to the gradual concentrations (CV). In the skin, the metallic perturbation caused a shift to an alternative stable state, leading to an incomplete recovery and therefore, facilitating the proliferation of opportunistic pathogens (like *Flavobacterium*). In the gut, the functional and phylogenetic diversity measurements suggest a complete community recovery in the CC group and gradual recovery in the CV group. The selective pressure exerted by cadmium on host and water microbiota may have left adaptive evolution patterns conserving functional diversity at the expense of taxonomic diversity. In both skin and gut microbiota, the recovery was associated with a significant increase of evenness and richness in the skin and vice versa in the gut. In the control group, as expected, the significant divergence from the initial community structure confirms the dynamic of bacterial strains through the developmental stage of fish juveniles. Consequently, community recovery was affected by both cadmium pressure and host development. In addition, our results have shown that the microbial assembly rules during the community recovery were both orchestrated by neutral and deterministic processes. In the water, community recovery was driven by a substantial role of phylogenetic structuring resulting from a combined pattern of stochasticity and cadmium-induced selective pressure, in which the causality remains unknown. Further studies are needed to quantify the interactions of neutrality and determinism in driving post-disturbance assembly of the host-associated microbiome during recovery.

## Supplementary information

**Additional file 1: Figure S1.** Dynamic of alpha-diversity divergence between host and water communities. The significant ANOVA results of alpha diversity between water (W), Skin(S) and Gut (Gut) communities in Control, CV and CC groups before and during disturbance, and after recovery period are represented with asterisks on the boxplots (0.001 : “***”, 0.01 : “**”, 0.05 : “*”).

**Additional file 2: Figure S2.** Beta-diversity divergence at the treatment level. This file combines all the NMDS (non-metric Multi-Dimensional Scaling) plots showing first two dimensions in the ordination of when using generalized Unifrac distance measure of water and host-microbial communities. The NMDS plots and PERMANOVA revealed a significant separation between different treatments and control (for the pairwise, see Table 4 for adjusted p-values after Benjamini-Hochberg correction in PERMANOVA and MRPP tests) at T0, T3, and T5 for skin and gut microbiota, and at T0, T3, TR1-TR4, and T5 for water microbial communities.

**Additional file 3: Figure S3.** Beta-diversity divergence at the community level. This file combines all the NMDS (non-metric Multi-Dimensional Scaling) plots and phylograms based on generalized Unifrac distances between water and host-microbial communities. The NMDS plots and PERMANOVA revealed a significant separation of among all type of communities per time (T0, T3, and T5) and treatment (Control, CC, CV).

**Additional file 4: Figure S4.** Heatmaps of cadmium with taxa diversity and composition in host and water communities. The correlations indicate a gradient from positive (blue) to negative (red) along a colour gradient, with rows representing diversity measures (richness, evenness) as well as cadmium concentrations, and columns indicating taxonomic levels. The gut microbiome in the constant CdCl_2_ (CC) and variable CdCl_2_ (CV) regimes showed a negative correlation between *Mycoplasma* and diversity indices. A strong positive correlation between *Actinomycetales* is noticeable in the CV. For the skin microbiome, not only *Actinomycetales*, but also *Burkholderiales*, and *Chromatiales* showed strong positive correlations with CV. This figure was produced using the Rhea package.

**Additional file 5: Figure S5.** Heatmaps of cadmium with taxa diversity and composition in water during the recovery period. The correlations indicate a gradient from positive (blue) to negative (red) along a colour gradient, with rows representing diversity measures (richness, evenness) as well as cadmium concentrations, and columns indicating taxonomic levels. In the constant CdCl_2_ (CC) and variable CdCl_2_ (CV) regimes, correlations of cadmium with taxa abundance showed variable profiles over time. This figure was produced using the Rhea package.

**Additional file 6: Figure S6.** Heat trees and stacked bar plots of water and host microbiome structure. This figure summarizes pairwise comparison of the community composition of water and each of the host communities for different treatments (Ctrl, CC and CV). Additionally, stacked bar plots of relative abundance at phylum level are provided for each community (water, skin, gut). The non-grey coloring (which category the branches are upregulated in) indicates significant differences in terms of log median ratios for samples from different habitats (Gut, Skin and Water) as determined by a Wilcox rank-sum test followed by a Benjamini-Hochberg (FDR) correction for multiple testing. The heat trees were built using metacoder and stacked barplots were produced using the Rhea package.

**Additional file 7: Table S1.** Alpha-diversity dynamics over time and treatments. This statistical summary reveals richness or evenness changes over time (Tables 1 and 2) and between control and treatments (Table 3) in water and host communities. The significant changes of alpha-diversity indices between treatments and control were statistically tested using Kruskal-Wallis and Wilcoxon tests by applying Benjamini-Hochberg correction. The same statistics were used to compare alpha-diversity over time.

**Additional file 8: Table S2.** Statistical summary of taxa divergence between treatments and control after recovery. This table summarises significant taxonomic changes in water and host-microbial communities between control and treatments using the Fisher test, and by applying Benjamini-Hochberg correction.

**Additional file 9: Tables S3.** Statistical summary of taxa divergence over time in host and water microbial communities after recovery**.** These tables summarise significant taxonomic changes over time in water and host-microbial communities using Kruskal-Wallis and Wilcoxon tests, and by applying Benjamini-Hochberg correction.

**Additional file 10: Tables S4.** Statistical summary of differential abundance between water and host microbial communities at all taxonomic levels. These tables summarise significant taxonomic changes between water and each of the host communities (skin and gut) at times T0 (sheet1), T3 (sheet2), and T5 (Sheet3) using Kruskal-Wallis and Wilcoxon tests, and by applying Benjamini-Hochberg correction.

**Additional file 11: Table S5.** Statistics of the neutral model in host and water microbiomes. This table summarises the neutral model fit based on the following parameters; the migration rate (m.ci) within 95% of the confidence interval, the goodness of fit(R^2^), number of samples, richness, abundance cutoff, percentage of % neutral OTUs and non-neutral OTUs.

**Additional file 12: Table S6.** List of OTUs that accounted for those that did not fit the neutral model.

**Additional file 13: Table S7.** List of OTUs that accounted for those that did not fit the neutral model and were assigned to *Mycoplasma* species.

## Data Availability

Sequencing data are available in the Sequence Read Archive (SRA) database at NCBI under the BioProject ID PRJNA556617

## Abbreviations

16 s rDNA: 16S ribosomal DNA
ANOVA: Analysis of variance
BEB: Back extraction buffer
BH: Benjamini-Hochberg correction test
CC: Cadmium constant concentration
CdCl2: Cadmium salts
CPAUL: Comités de Protection des Animaux de l’Université Laval
Ctrl: Regime of negative control
CV: Cadmium variable concentration
FDR: False discovery rate
MRPP: Multiple response permutation procedure
NLS: Non-linear least squares model
NMDS: Non-metric multi-dimensional scaling
OTUs: Operational taxonomic units
PCR: Polymerase chain reaction
PERMANOVA: Permutational analysis of variance
ppb: Parts per billion
RDP: Ribosomal Database Project
