# Supplementary figures and images for: Community recovery dynamics in yellow perch microbiome after gradual and constant metallic perturbations
Source: Microbiome. 2020 Feb 10;8:14. doi: 10.1186/s40168-020-0789-0 (PMC7011381; doi:10.1186/s40168-020-0789-0)

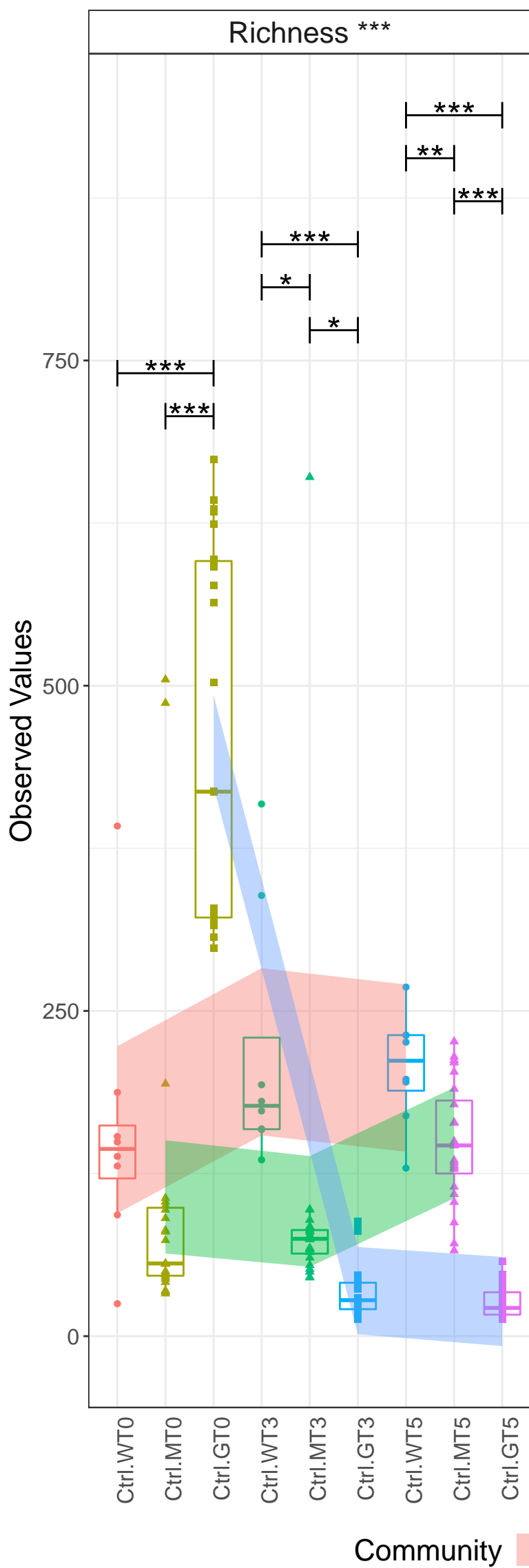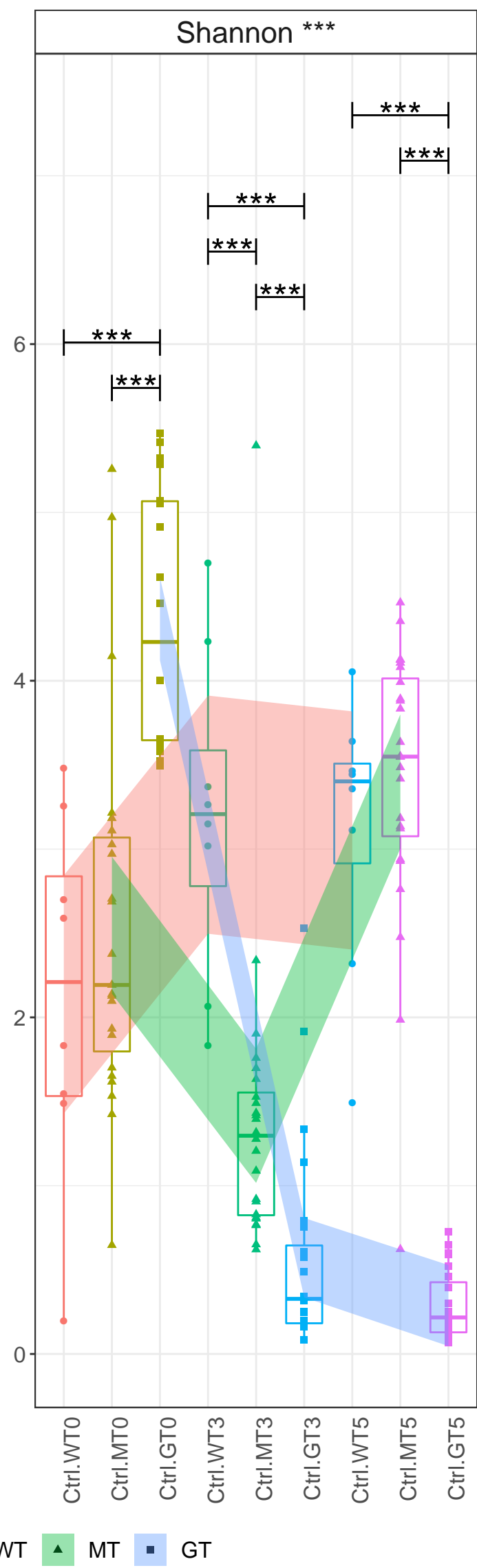

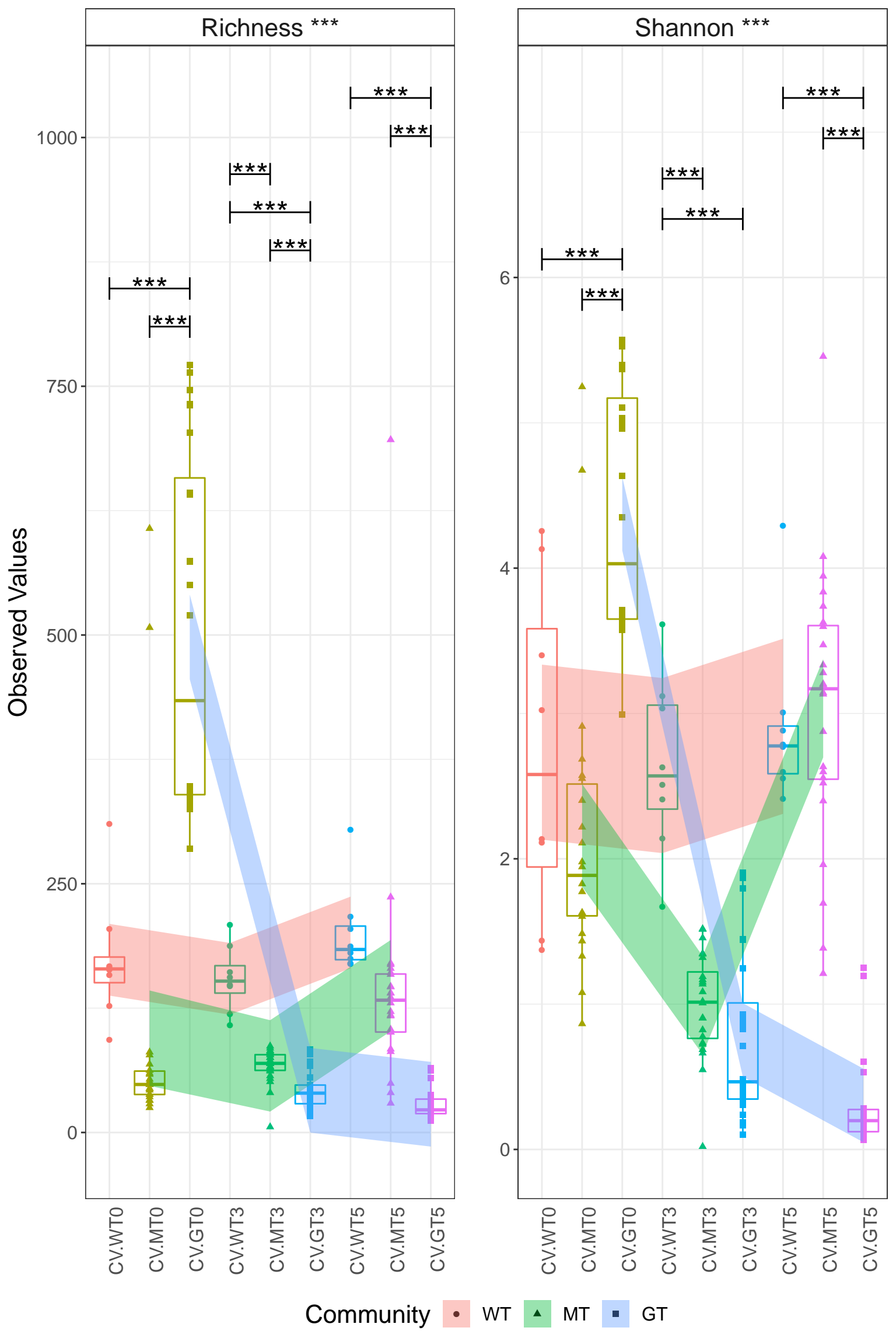

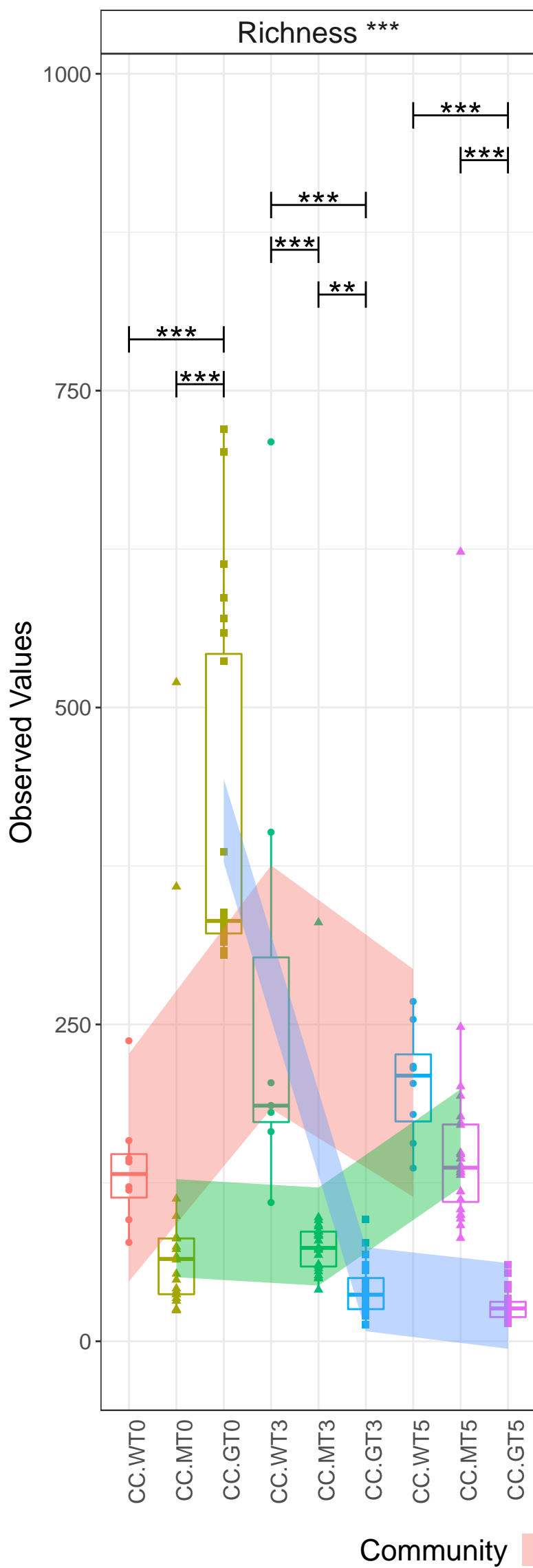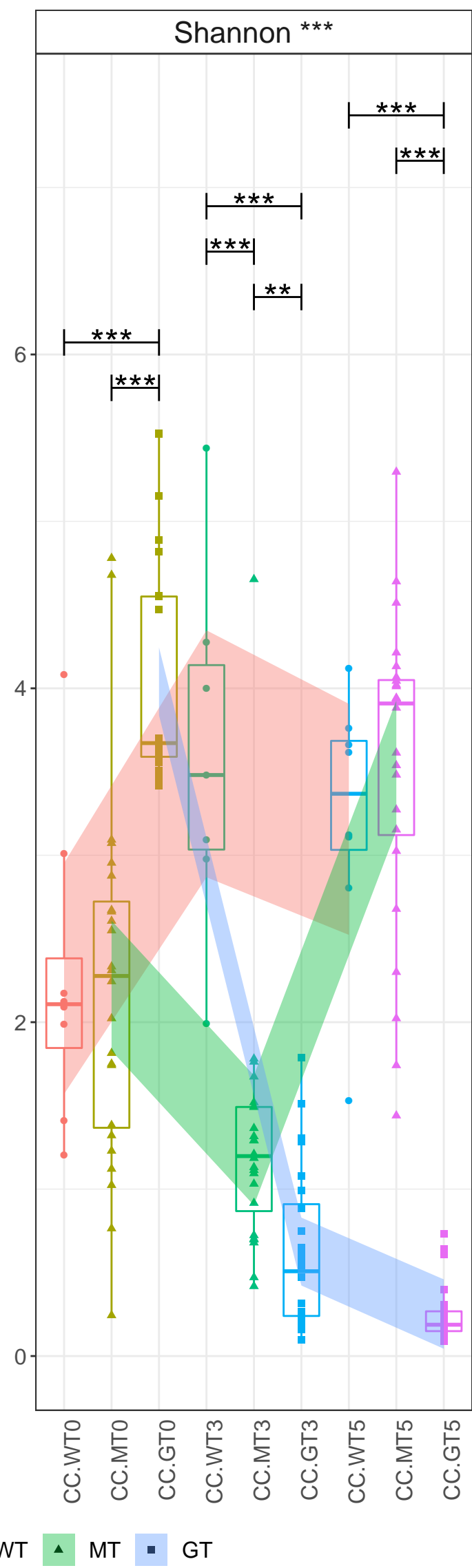

Supplement: Supplementary file 2 — Additional file 1: Figure S1. Dynamic of alpha-diversity divergence between host and water communities. The significant ANOVA results of alpha diversity between water (W), Skin(S) and Gut (Gut) communities in Control, CV and CC groups before and during disturbance, and after recovery period are represented with asterisks on the boxplots (0.001 : “***”, 0.01 : “**”, 0.05 : “*”). [file 40168_2020_789_MOESM1_ESM.pdf]

Gut-CC-T5

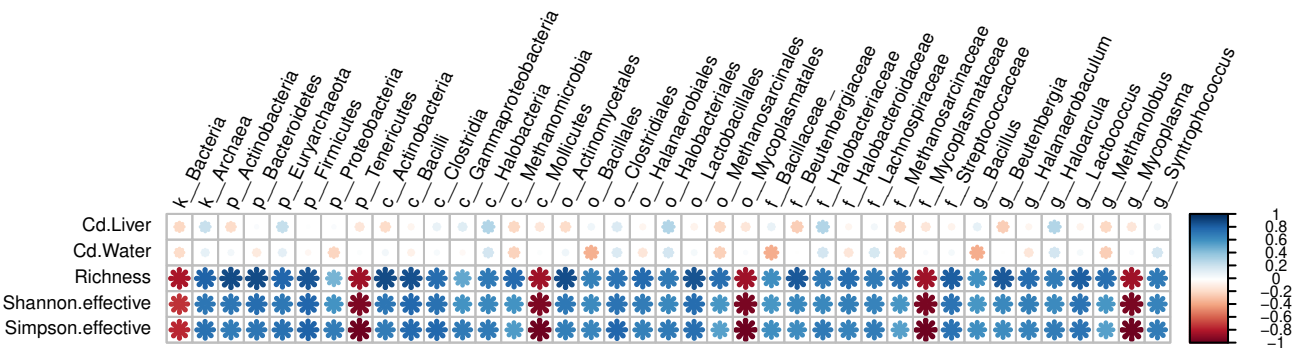

Gut-CV-T5

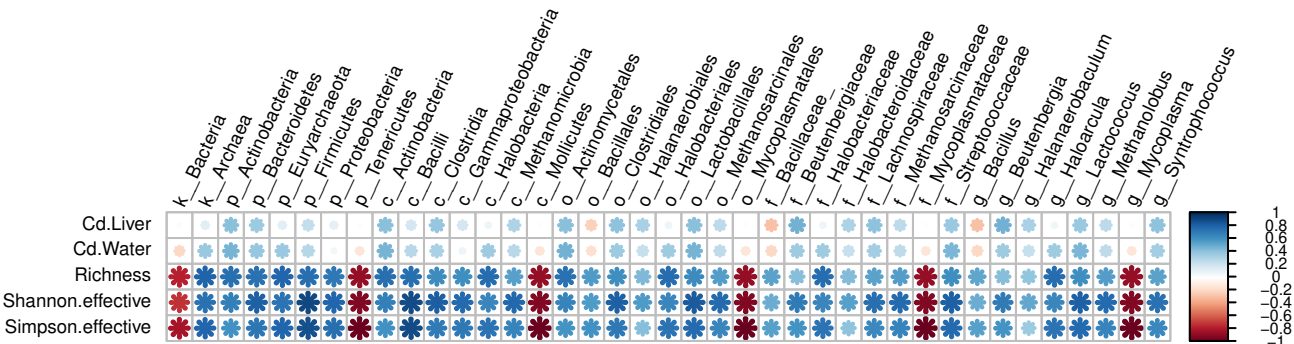

Skin-CC-T5

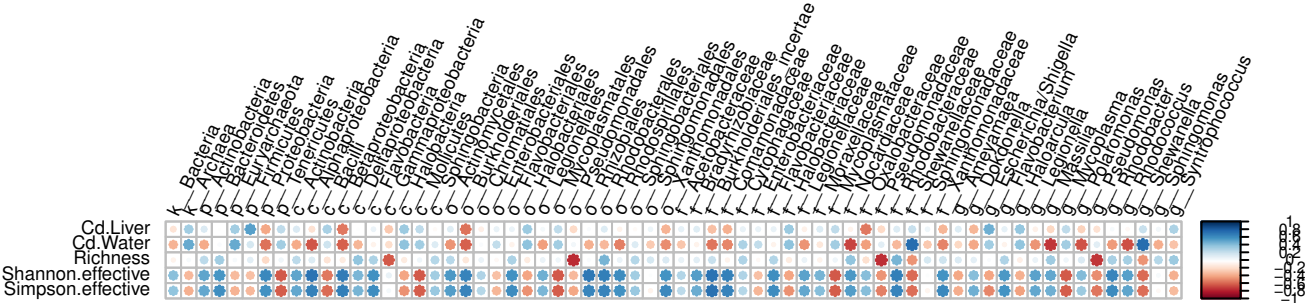

Skin-CV-T5

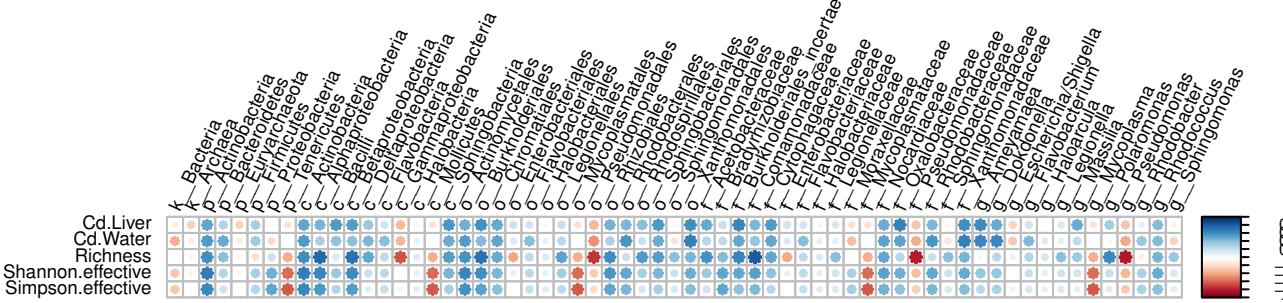

Water-CC-T5

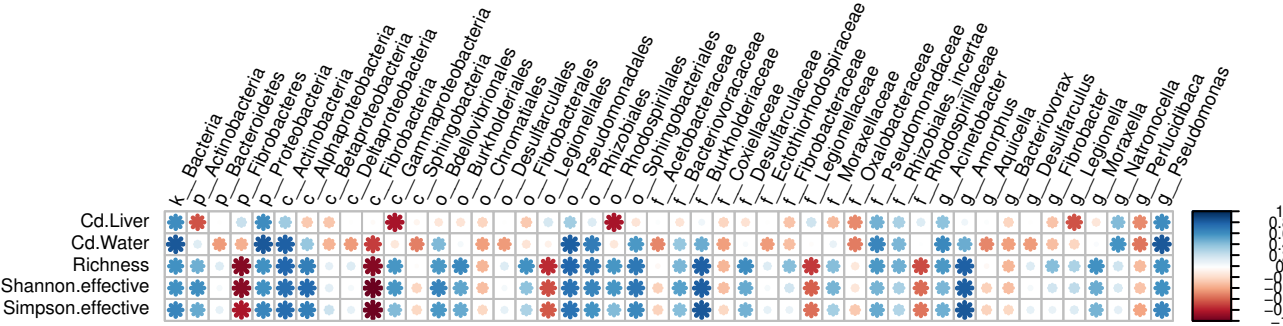

Water-CV-T5

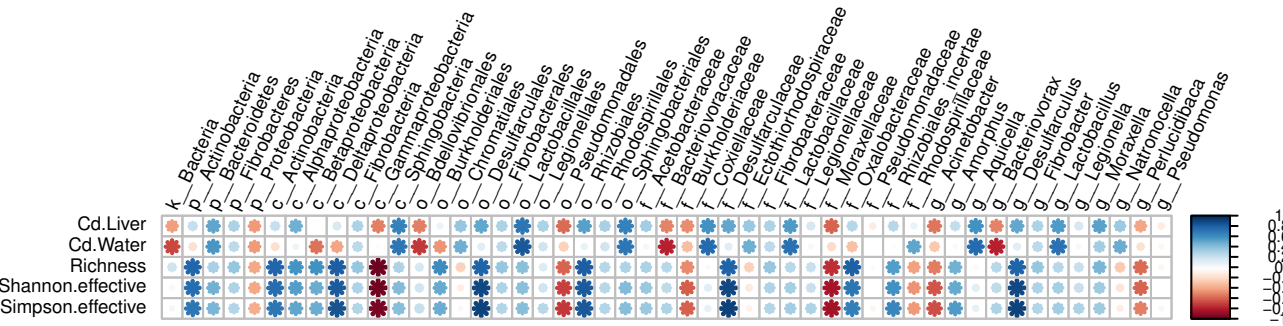

Supplement: Supplementary file 5 — Additional file 4: Figure S4. Heatmaps of cadmium with taxa diversity and composition in host and water communities. The correlations indicate a gradient from positive (blue) to negative (red) along a colour gradient, with rows representing diversity measures (richness, evenness) as well as cadmium concentrations, and columns indicating taxonomic levels. The gut microbiome in the constant CdCl2 (CC) and variable CdCl2 (CV) regimes showed a negative correlation between Mycoplasma and diversity indices. A strong positive correlation between Actinomycetales is noticeable in the CV. For the skin microbiome, not only Actinomycetales, but also Burkholderiales, and Chromatiales showed strong positive correlations with CV. This figure was produced using the Rhea package. [file 40168_2020_789_MOESM4_ESM.pdf]

TR1-CC

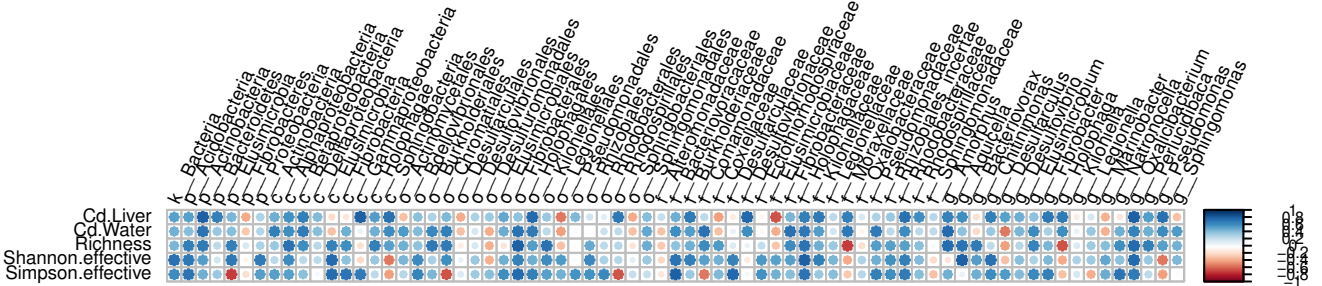

TR1-CV

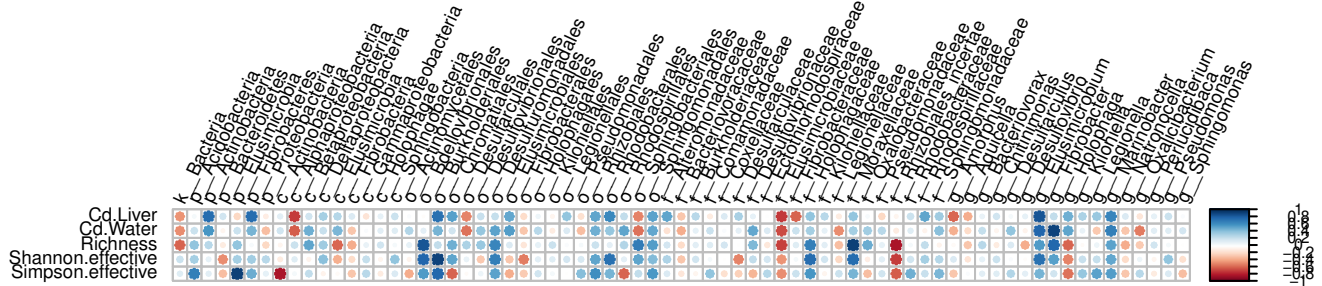

TR2-CC

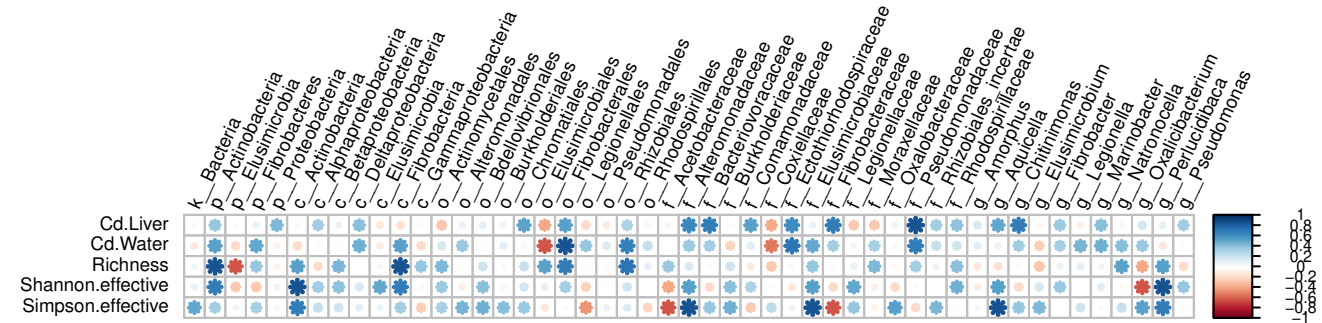

TR2-CV

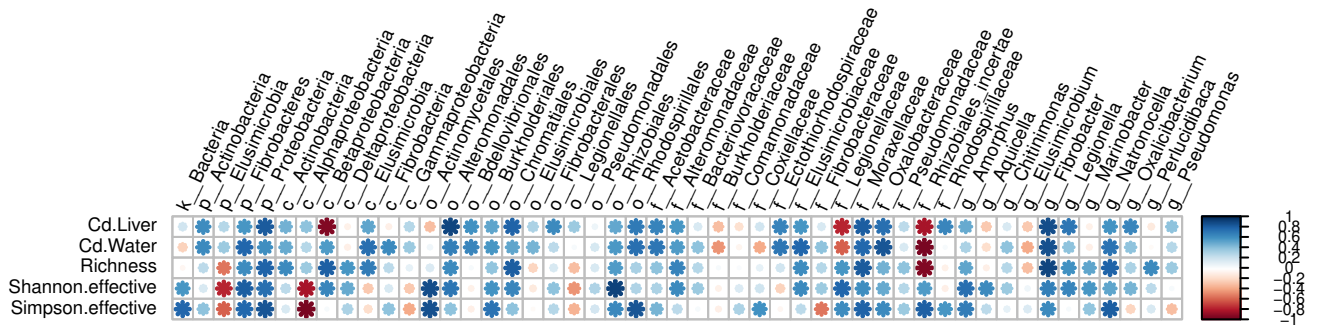

TR4-CC

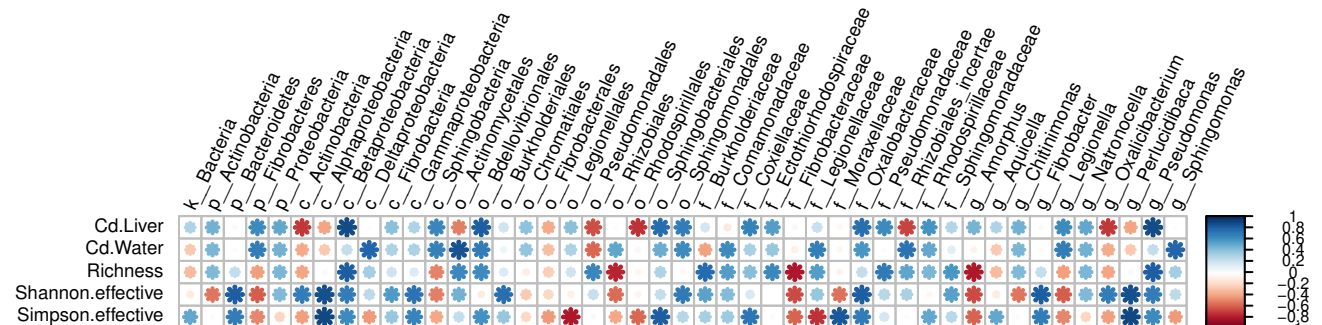

TR4-CV

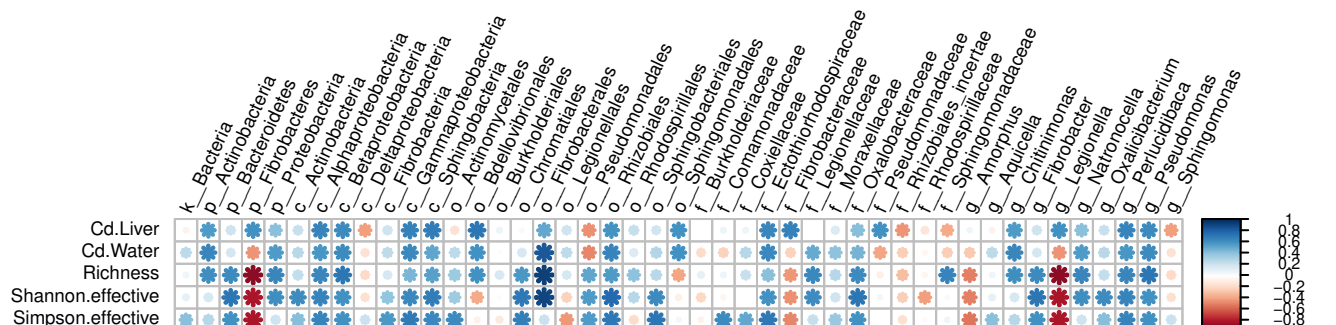

Supplement: Supplementary file 6 — Additional file 5: Figure S5. Heatmaps of cadmium with taxa diversity and composition in water during the recovery period. The correlations indicate a gradient from positive (blue) to negative (red) along a colour gradient, with rows representing diversity measures (richness, evenness) as well as cadmium concentrations, and columns indicating taxonomic levels. In the constant CdCl2 (CC) and variable CdCl2 (CV) regimes, correlations of cadmium with taxa abundance showed variable profiles over time. This figure was produced using the Rhea package. [file 40168_2020_789_MOESM5_ESM.pdf]

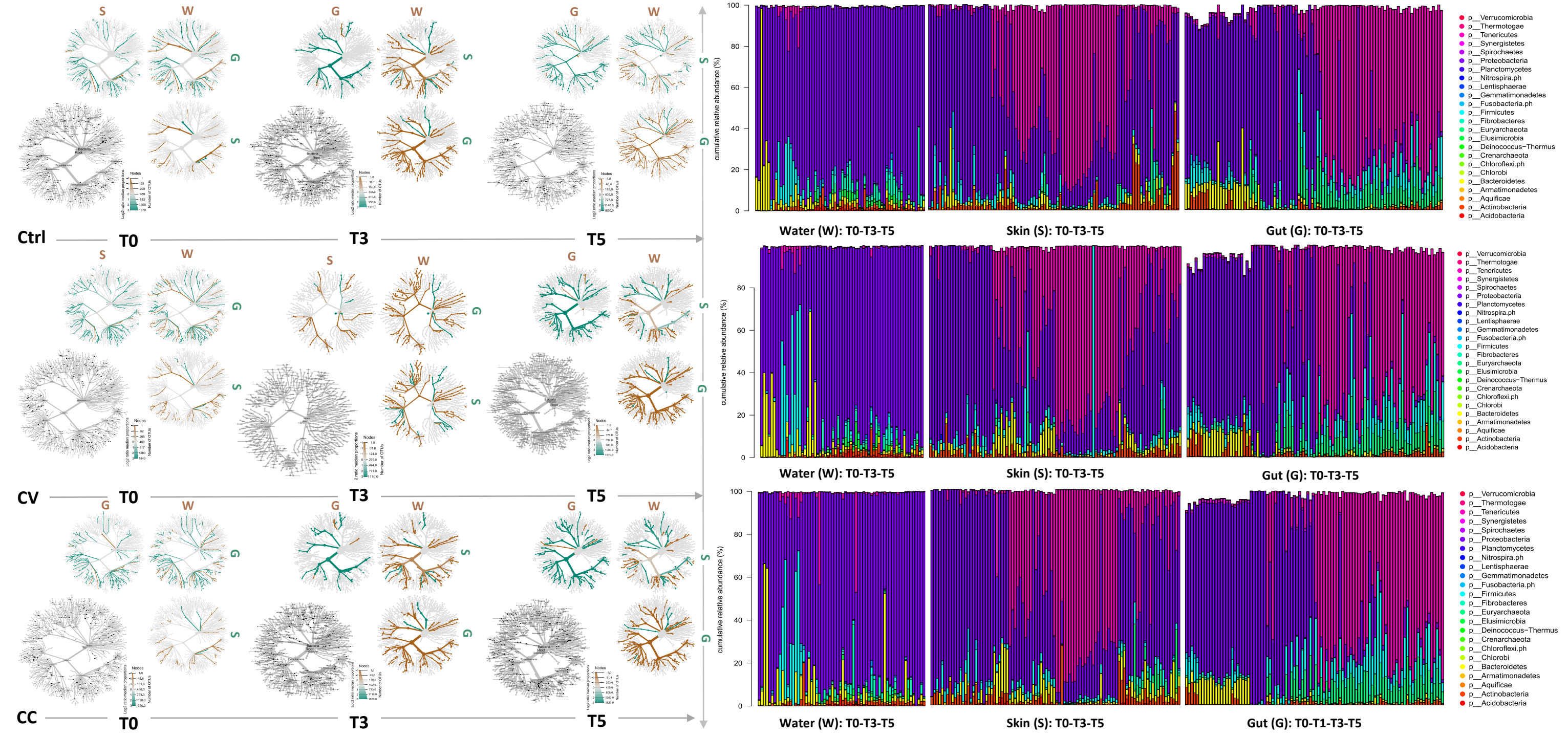

Supplement: Supplementary file 7 — Additional file 6: Figure S6. Heat trees and stacked bar plots of water and host microbiome structure. This figure summarizes pairwise comparison of the community composition of water and each of the host communities for different treatments (Ctrl, CC and CV). Additionally, stacked bar plots of relative abundance at phylum level are provided for each community (water, skin, gut). The non-grey coloring (which category the branches are upregulated in) indicates significant differences in terms of log median ratios for samples from different habitats (Gut, Skin and Water) as determined by a Wilcox rank-sum test followed by a Benjamini-Hochberg (FDR) correction for multiple testing. The heat trees were built using metacoder and stacked barplots were produced using the Rhea package. [file 40168_2020_789_MOESM6_ESM.pdf]
