# Supplementary material for: Community recovery dynamics in yellow perch microbiome after gradual and constant metallic perturbations
Source: Microbiome. 2020 Feb 10;8:14. doi: 10.1186/s40168-020-0789-0 (PMC7011381; doi:10.1186/s40168-020-0789-0)

The beta-diversity of water (W) communities at time T0

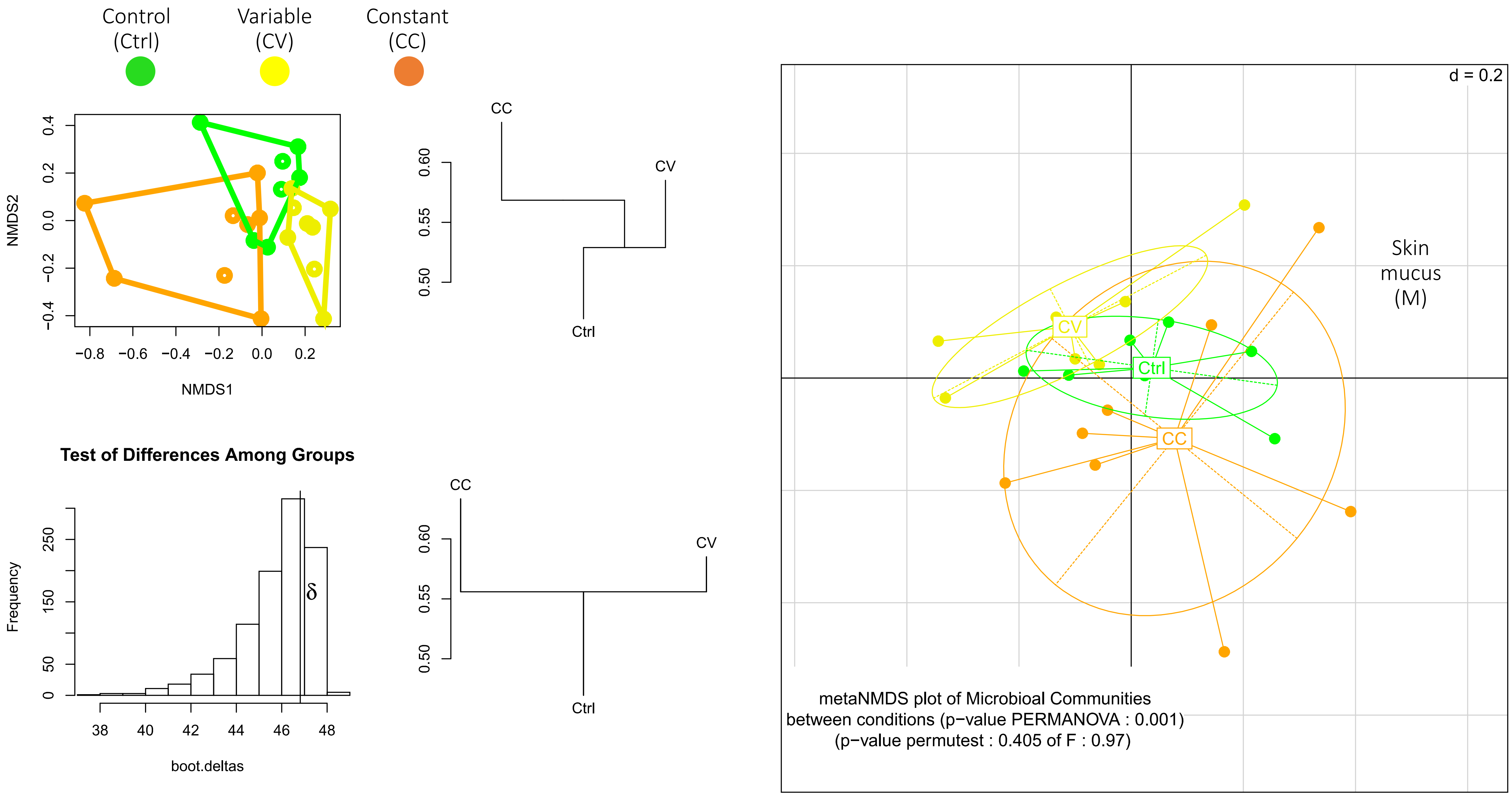

The beta-diversity of Water communities during cadmium exposure at time T3

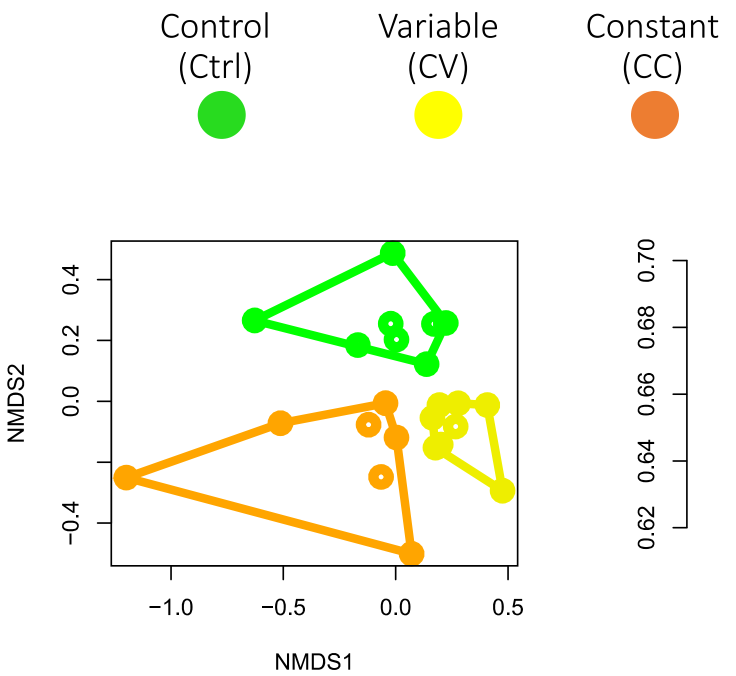

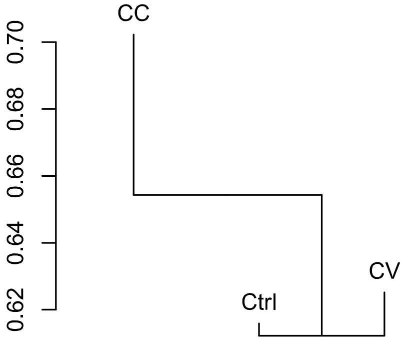

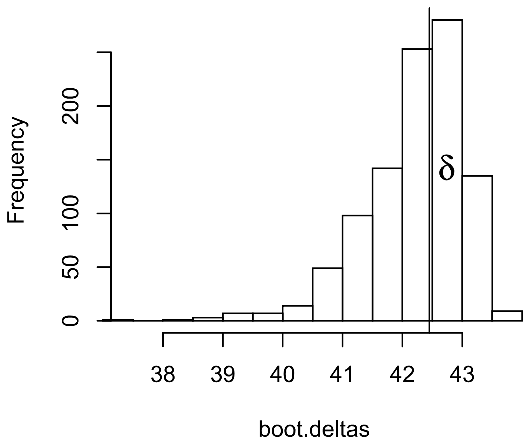

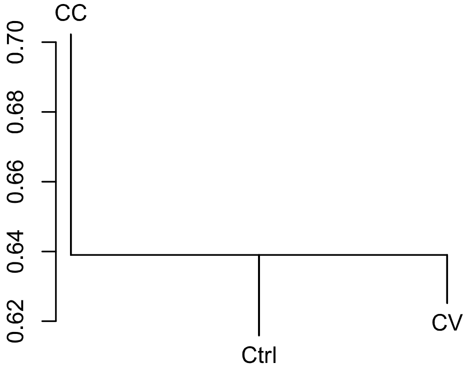

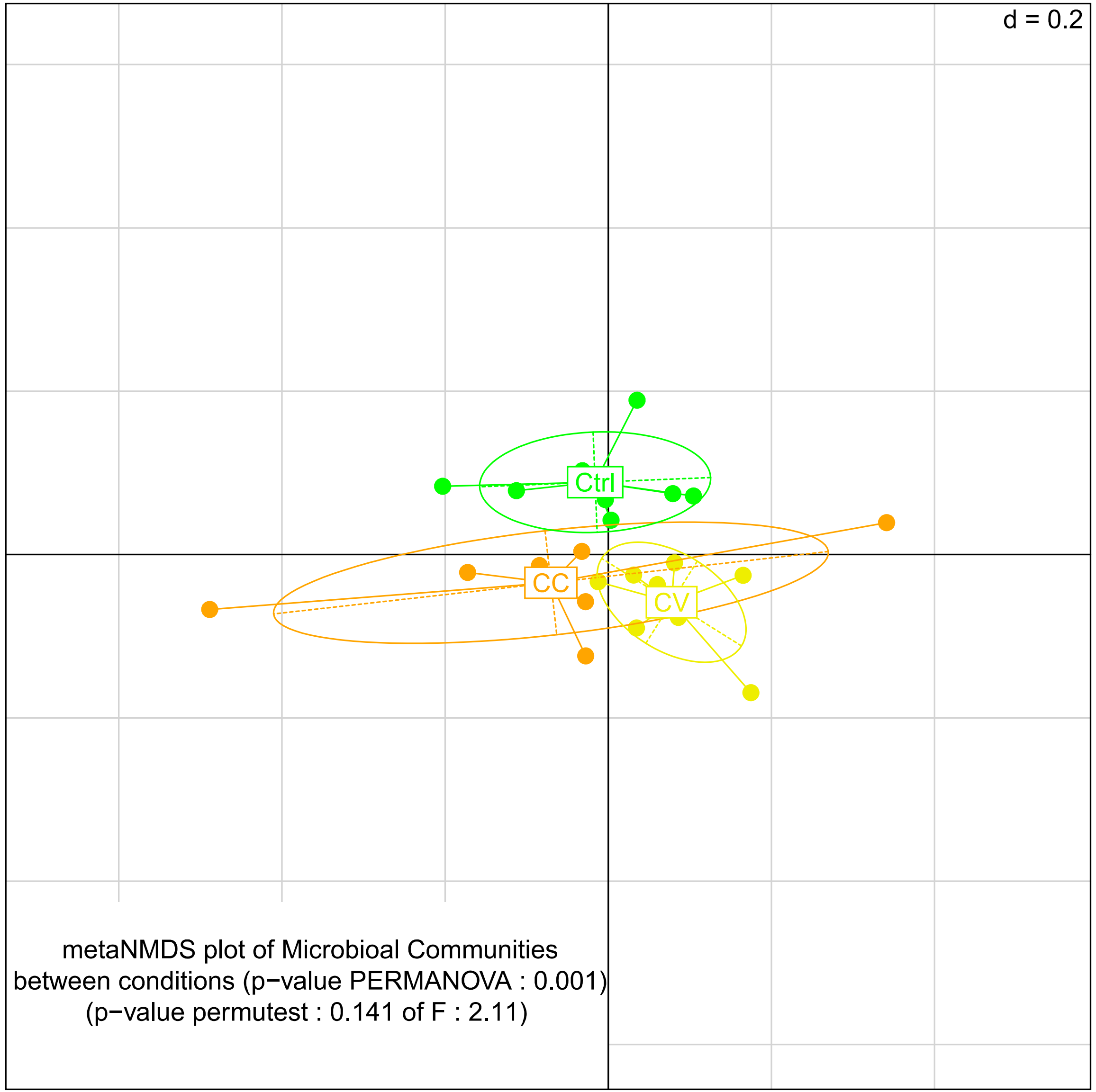

The beta-diversity of water (W) communities at recovery time TR1

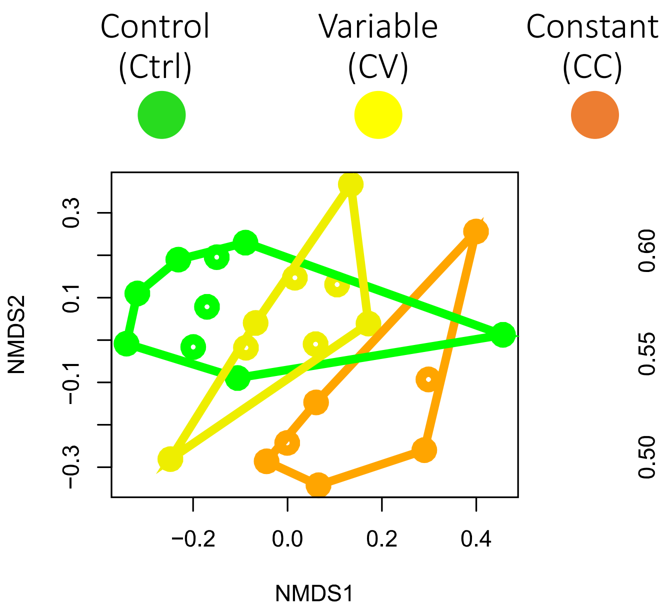

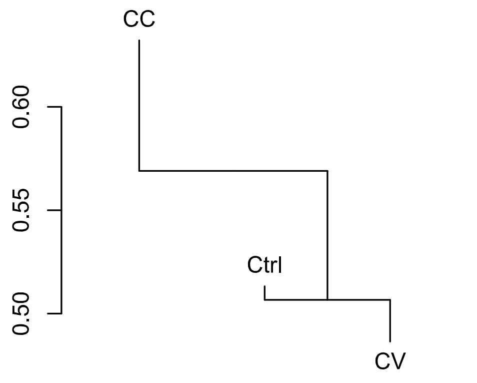

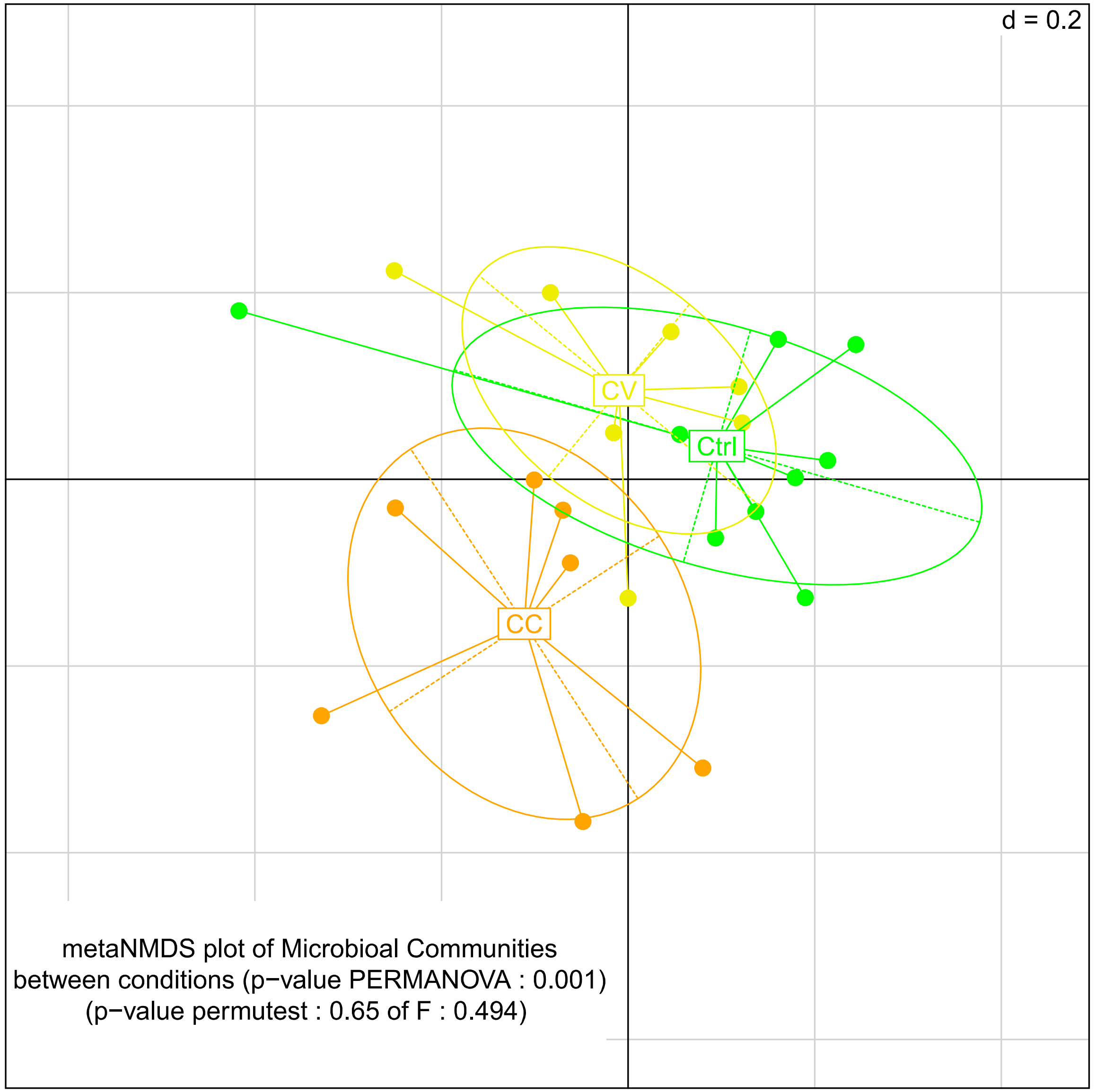

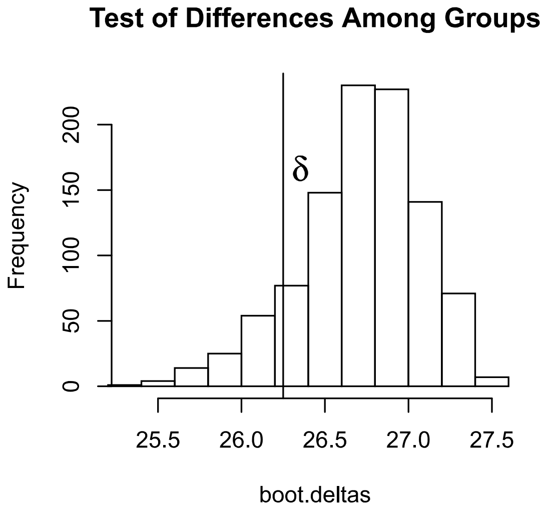

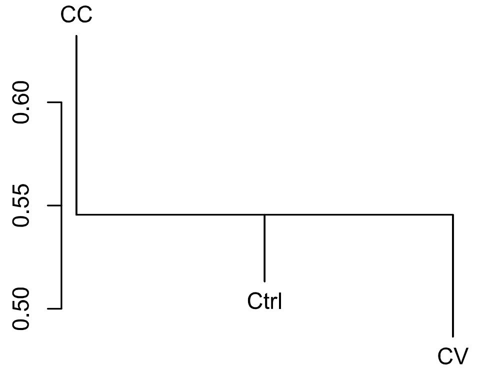

The beta-diversity of water (W) communities at recovery time TR2

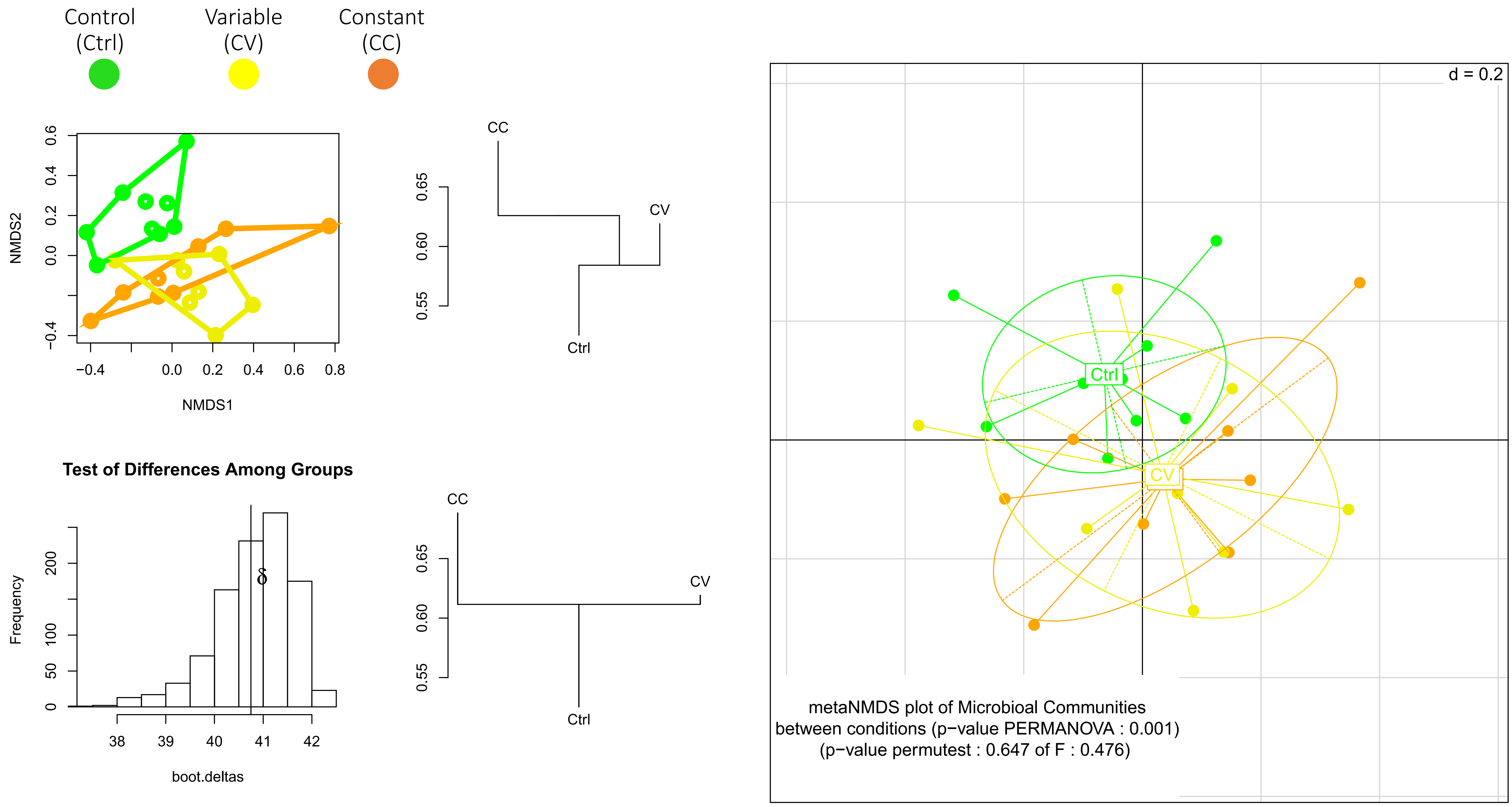

The beta-diversity of water (W) communities at recovery time TR3

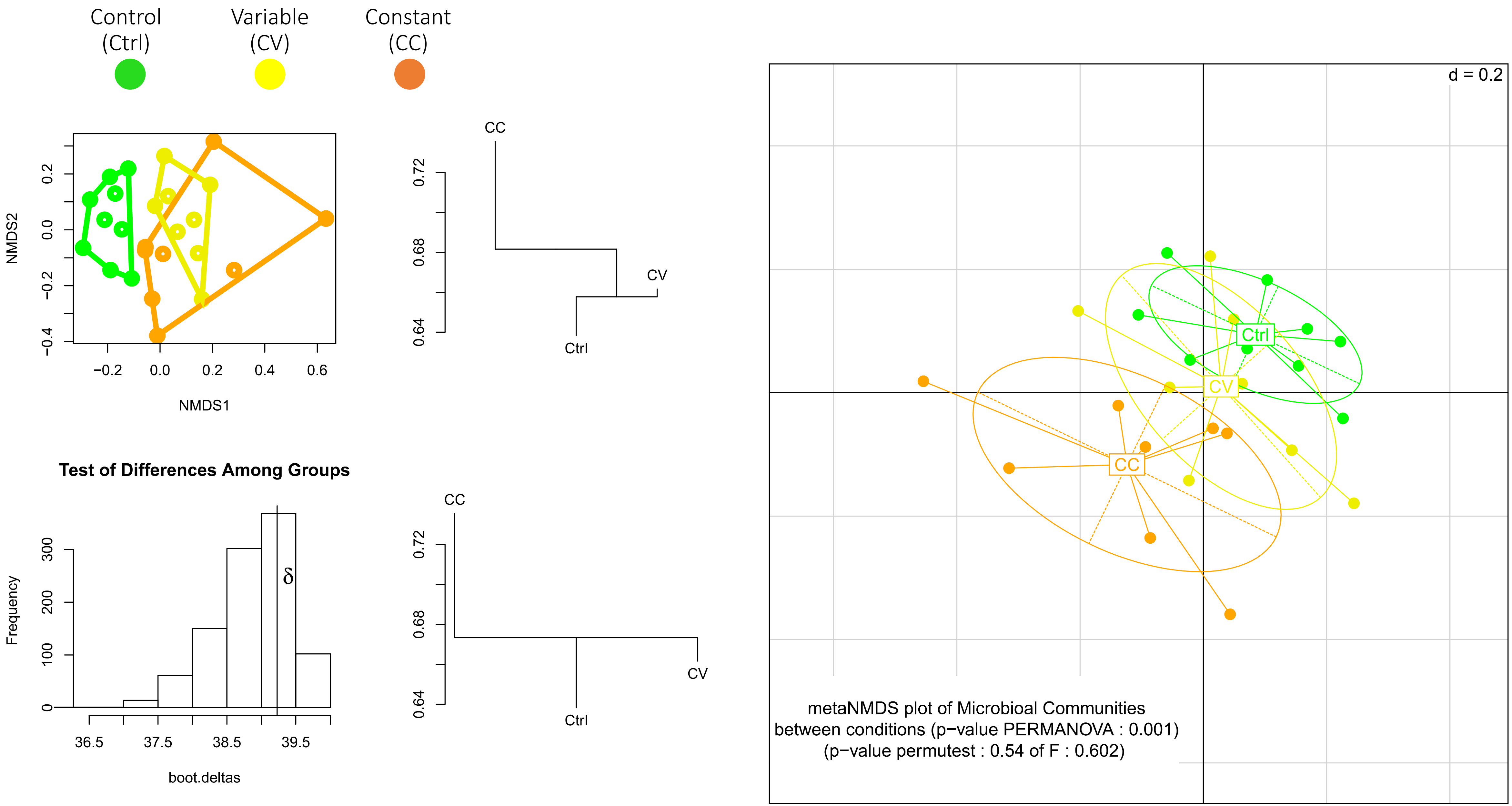

The beta-diversity of water (W) communities at recovery time TR4

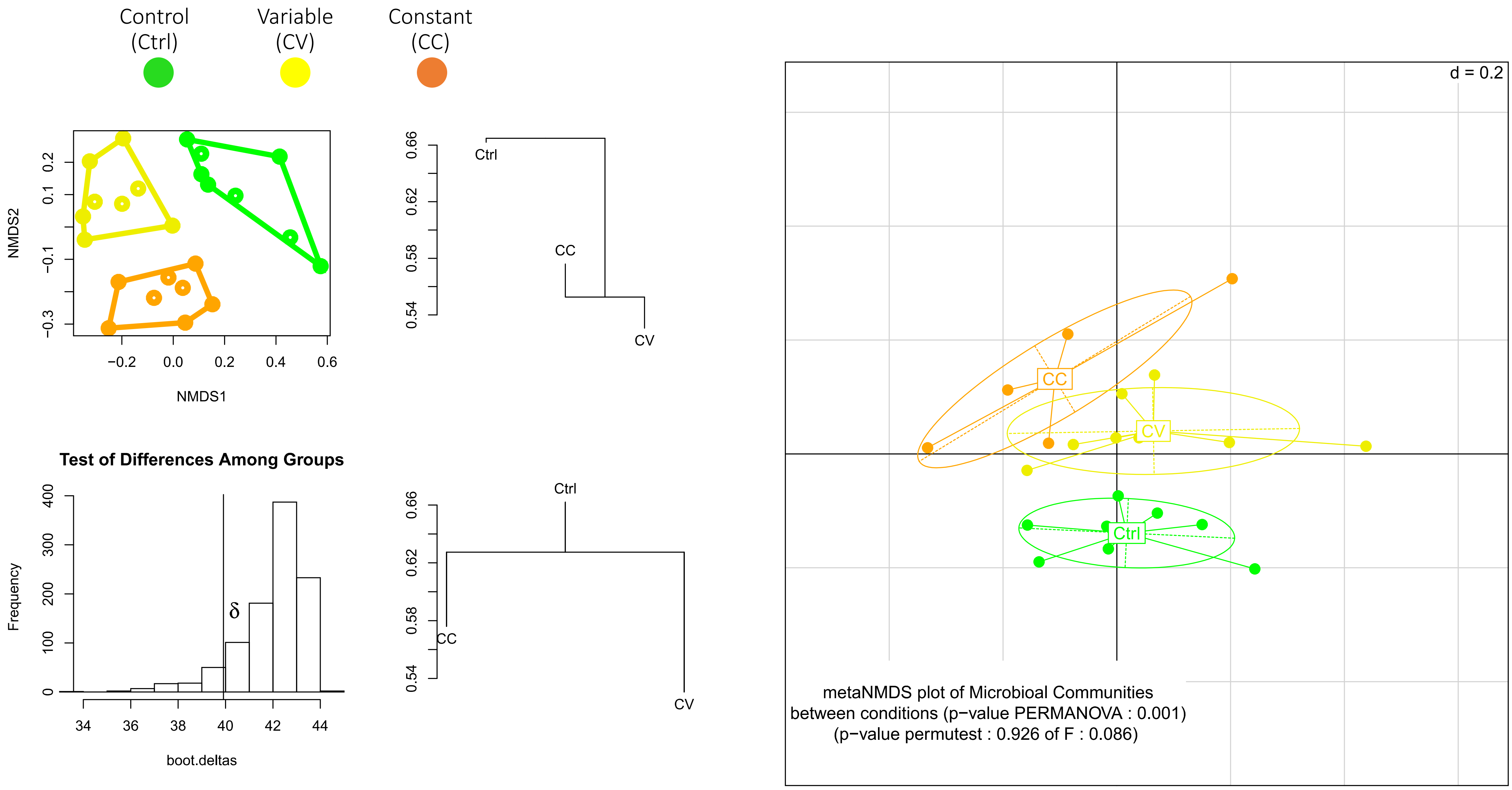

The beta-diversity of water (W) communities at the end of recovery time T5

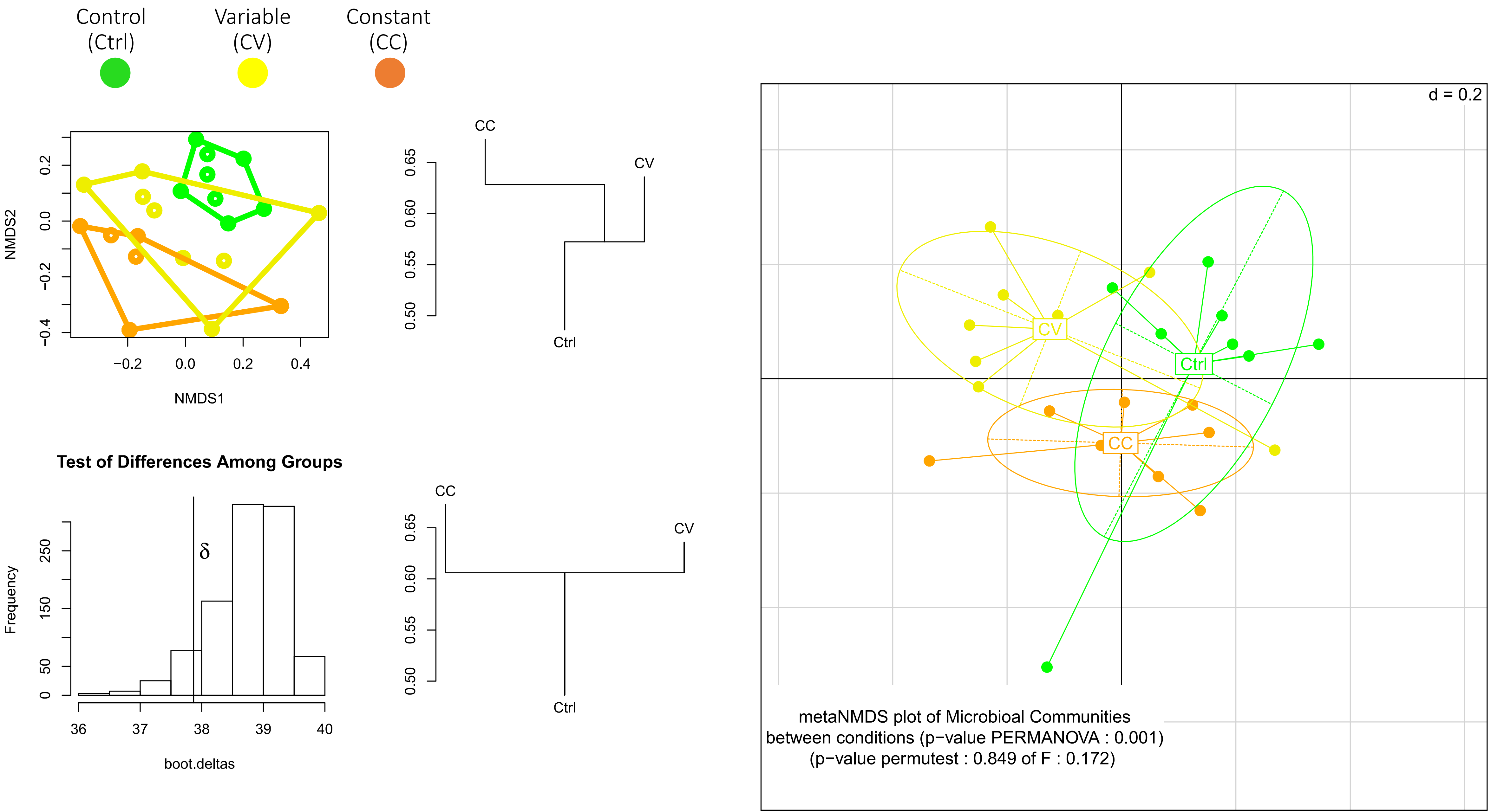

The beta-diversity of Skin communities before disturbance at time T0

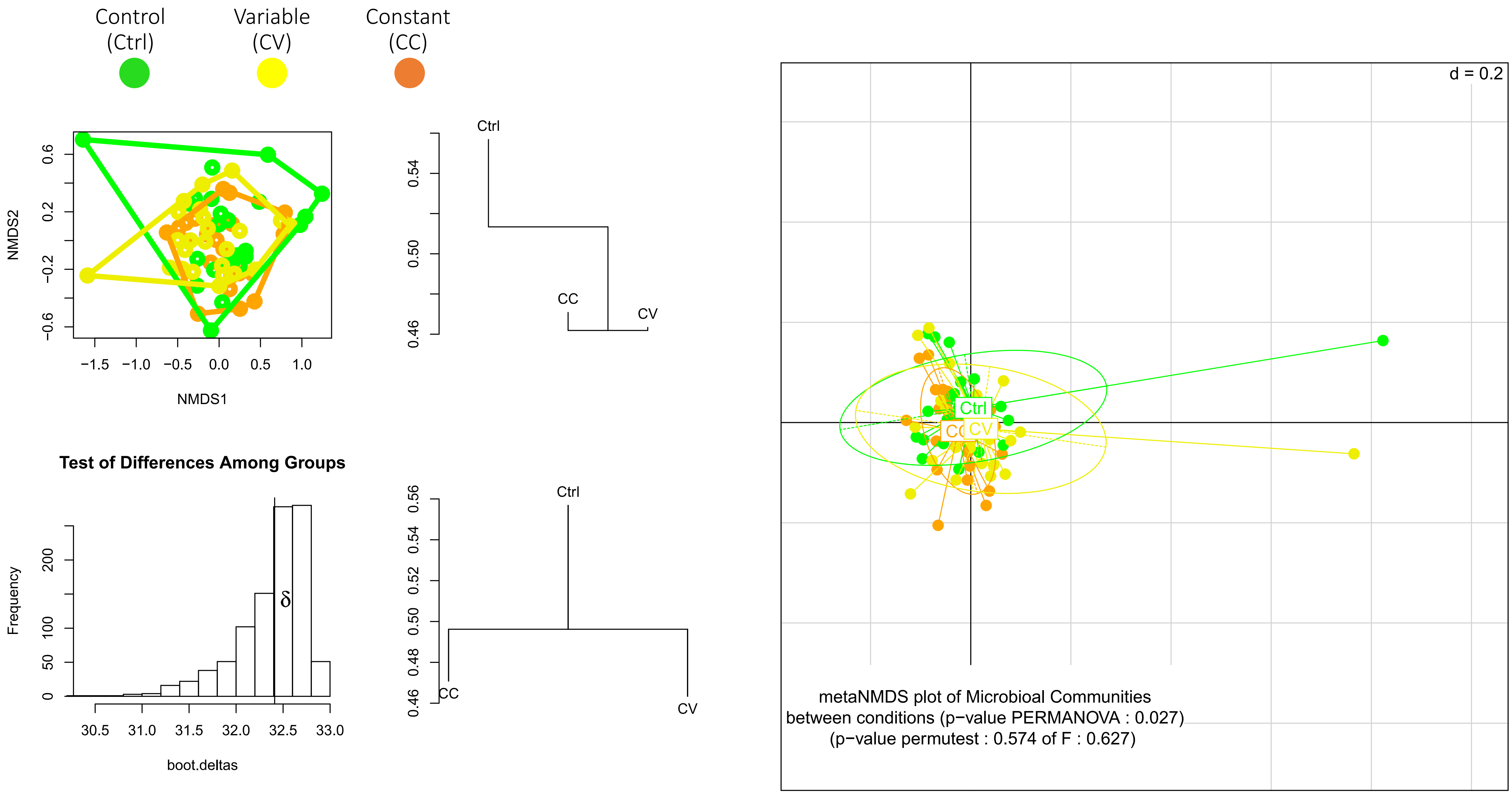

The beta-diversity of Skin communities during cadmium exposure at time T3

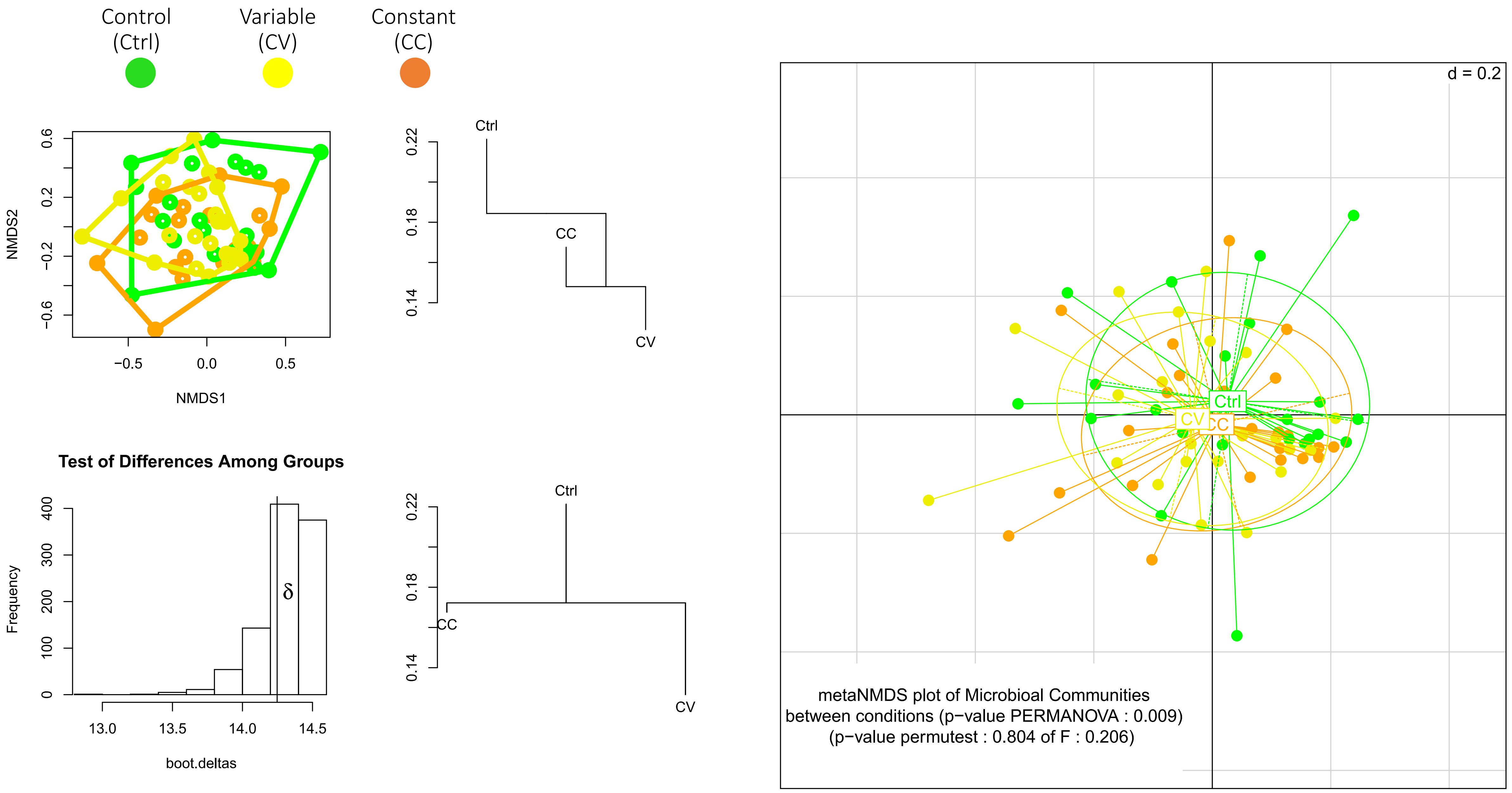

The beta-diversity of Skin communities at the end of recovery time T5

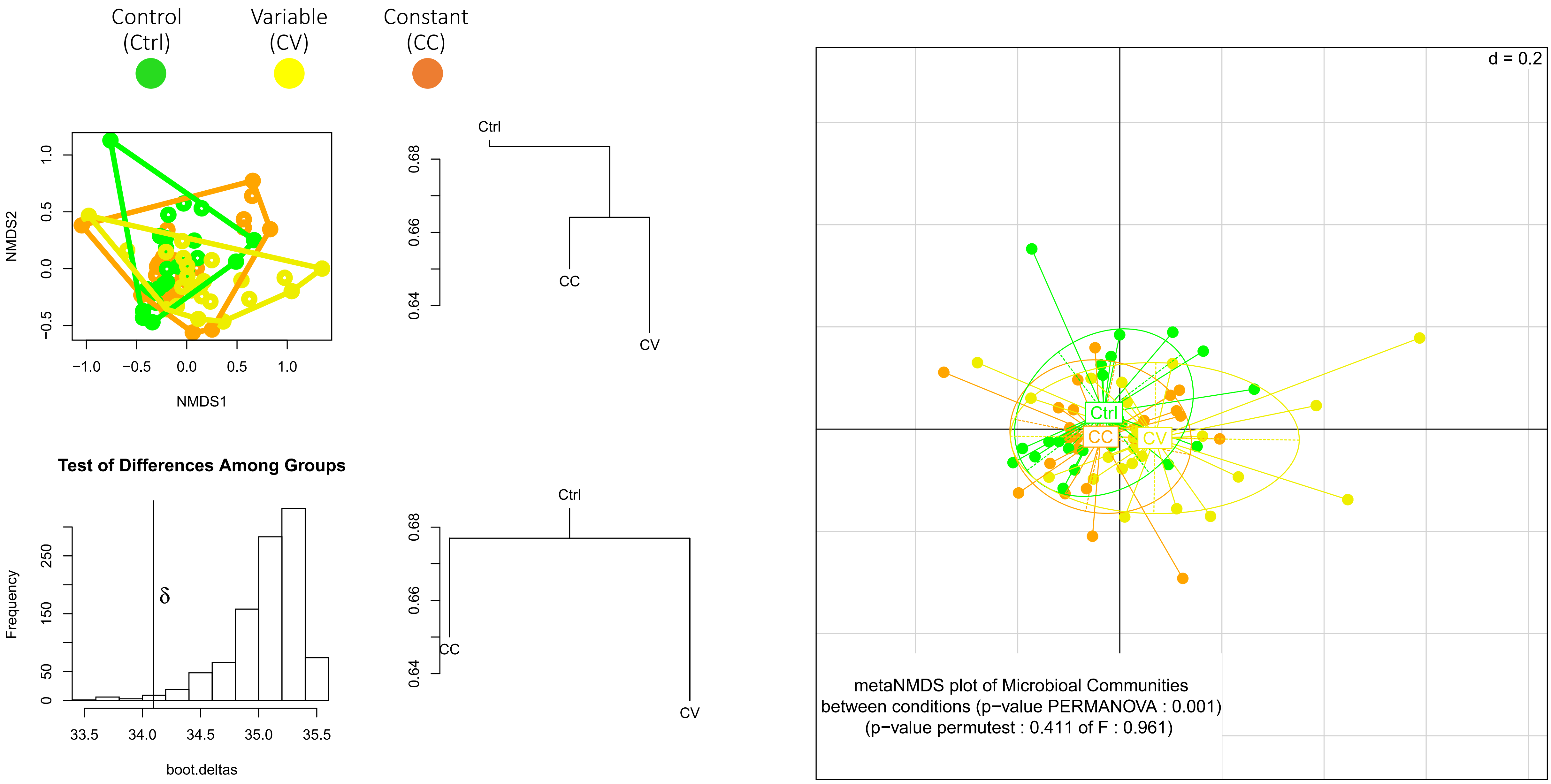

The beta-diversity of Gut communities before disturbance at time T0

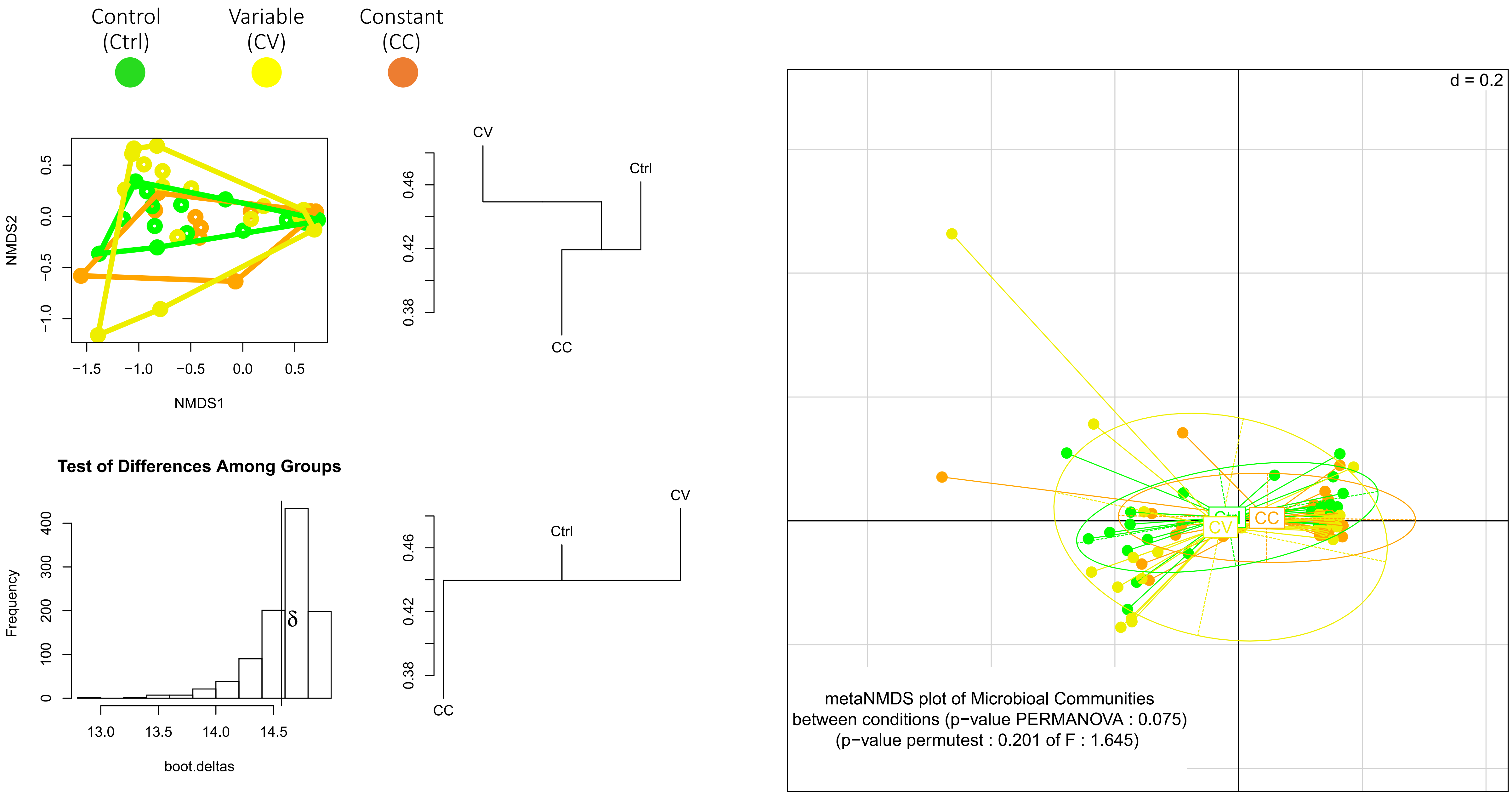

The beta-diversity of Gut communities during cadmium exposure at time T3

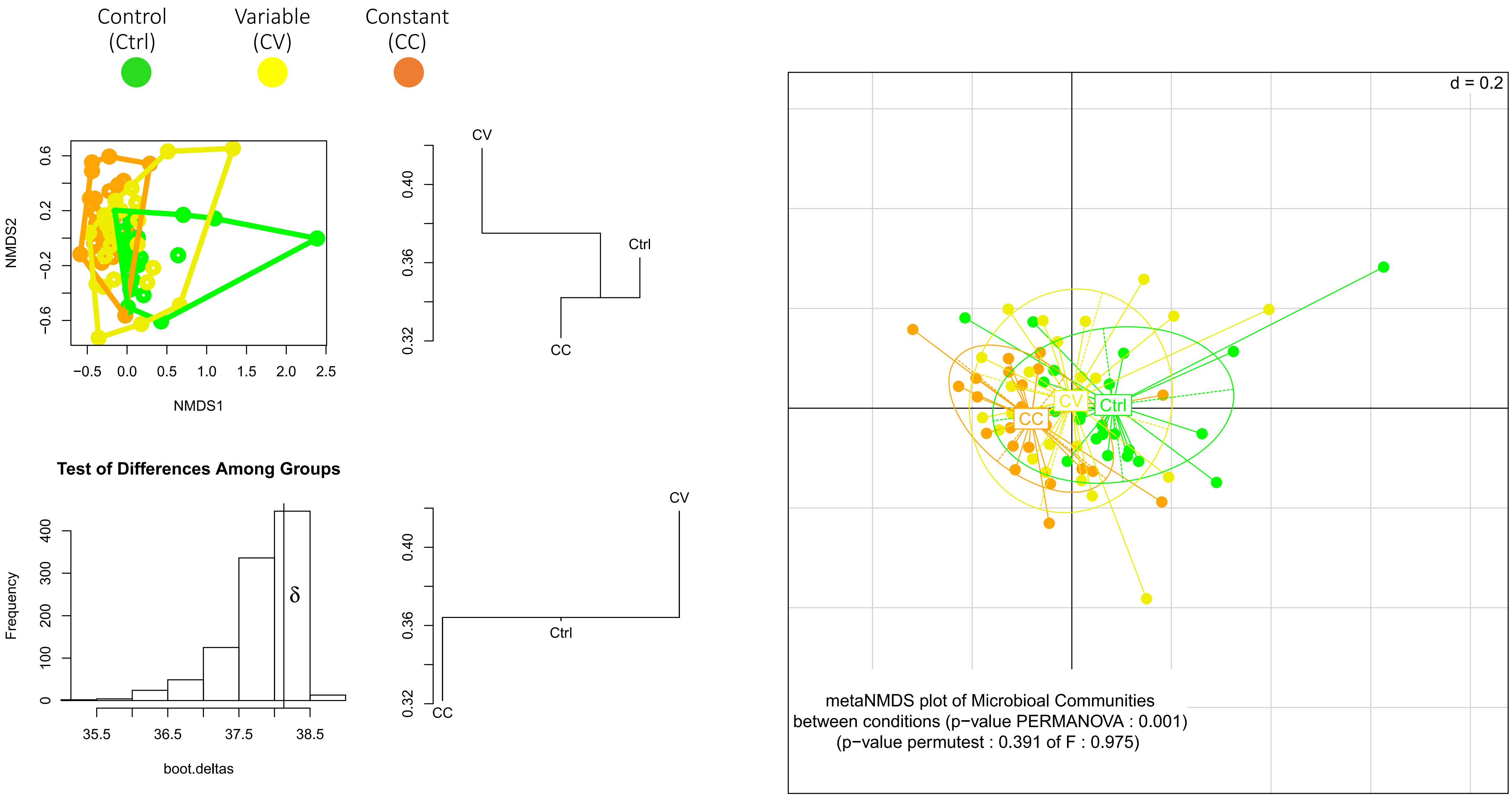

The beta-diversity of Gut communities at the end of recovery time T5

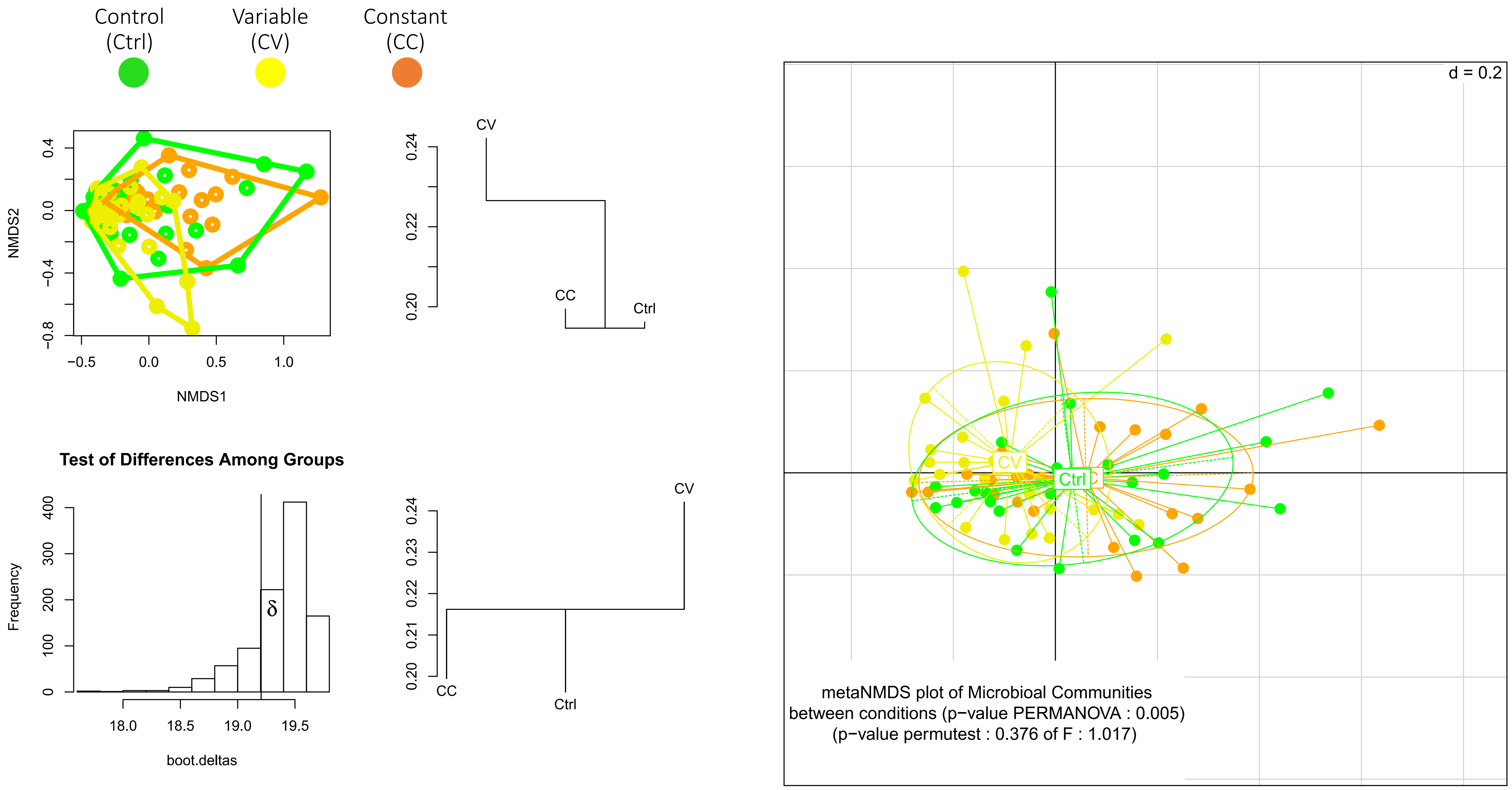

Supplement: Supplementary file 3 — Additional file 2: Figure S2. Beta-diversity divergence at the treatment level. This file combines all the NMDS (non-metric Multi-Dimensional Scaling) plots showing first two dimensions in the ordination of when using generalized Unifrac distance measure of water and host-microbial communities. The NMDS plots and PERMANOVA revealed a significant separation between different treatments and control (for the pairwise, see Table 4 for adjusted p-values after Benjamini-Hochberg correction in PERMANOVA and MRPP tests) at T0, T3, and T5 for skin and gut microbiota, and at T0, T3, TR1-TR4, and T5 for water microbial communities. [file 40168_2020_789_MOESM2_ESM.pdf]
