# Supplementary material for: Community recovery dynamics in yellow perch microbiome after gradual and constant metallic perturbations
Source: Microbiome. 2020 Feb 10;8:14. doi: 10.1186/s40168-020-0789-0 (PMC7011381; doi:10.1186/s40168-020-0789-0)
Supplement: Supplementary file 4 — Additional file 3: Figure S3. Beta-diversity divergence at the community level. This file combines all the NMDS (non-metric Multi-Dimensional Scaling) plots and phylograms based on generalized Unifrac distances between water and host-microbial communities. The NMDS plots and PERMANOVA revealed a significant separation of among all type of communities per time (T0, T3, and T5) and treatment (Control, CC, CV). [file 40168_2020_789_MOESM3_ESM.pdf]

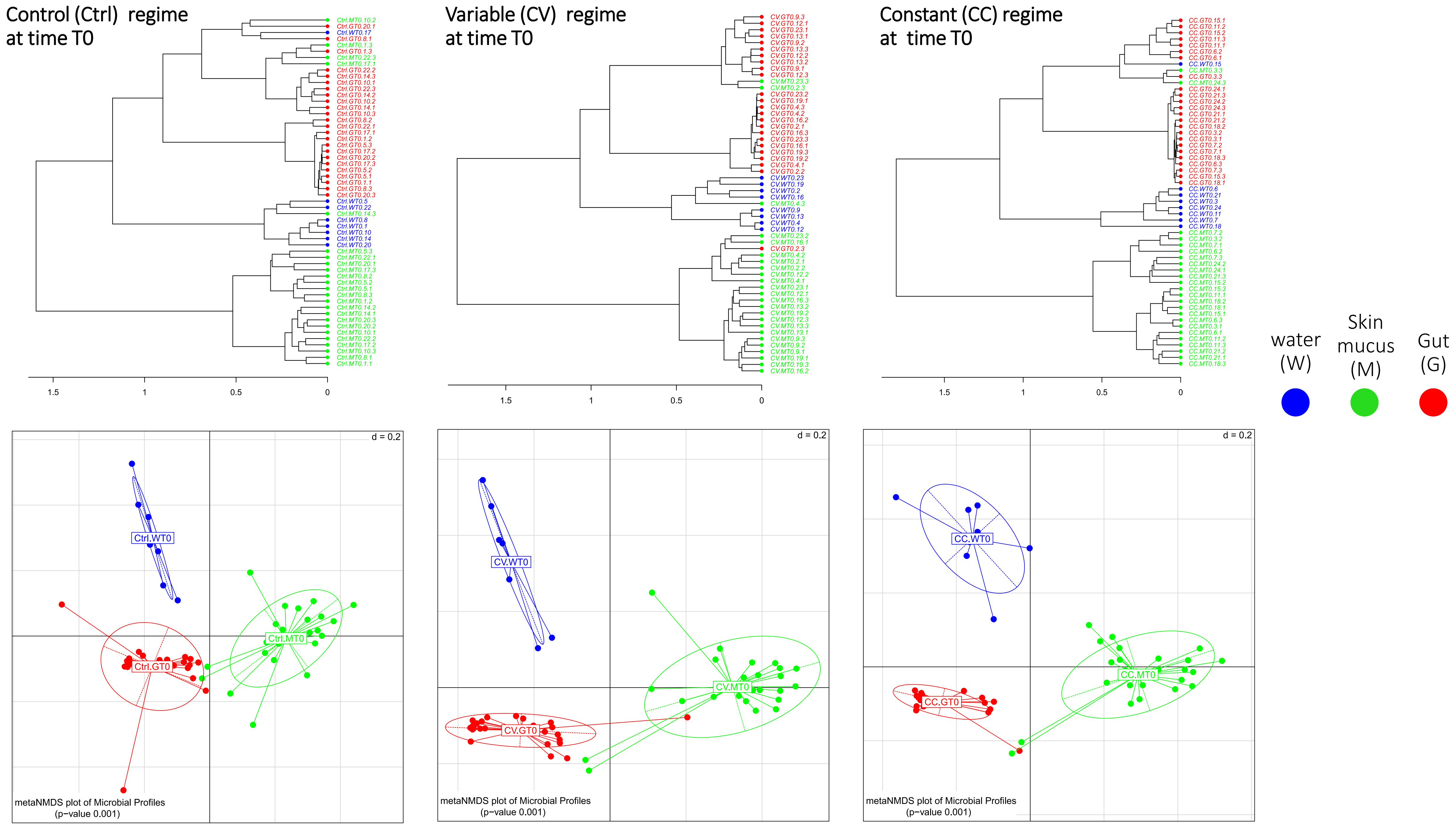

Control (Ctrl) regime
at time T0
Variable (CV) regime
at time T0
Constant (CC) regime
at time T0
1.5
1
0.5
0
water
(W)
Skin
mucus
(M)
Gut
(G)
d = 0.2
Ctrl.WT0
Ctrl.MT0
Ctrl.GT0
CV.WT0
CV.MT0
CV.GT0
CC.WT0
CC.MT0
CC.GT0
metaNMDS plot of Microbial Profiles
(p−value 0.001)

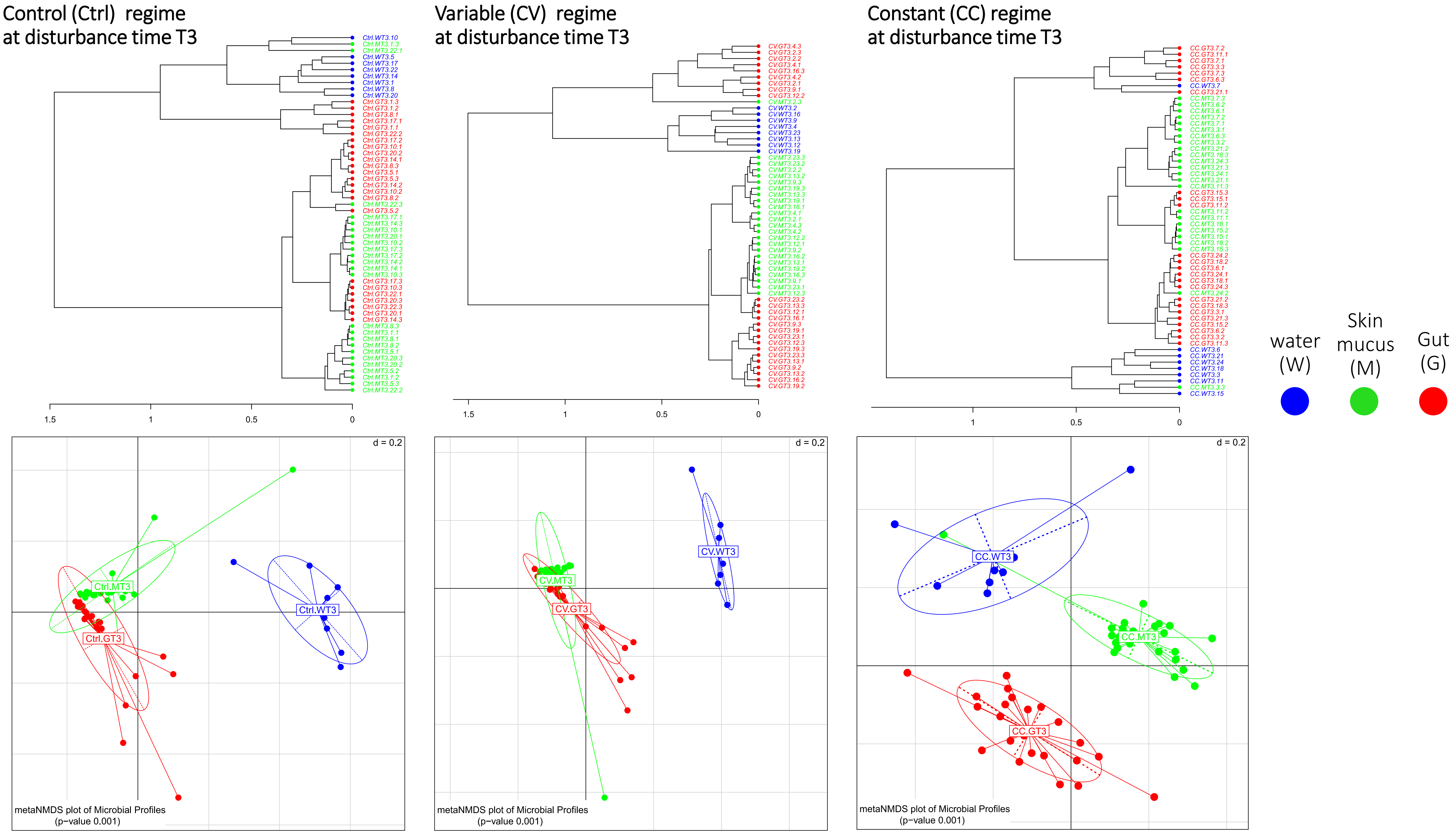
Control (Ctrl) regime
at disturbance time T3
Variable (CV) regime
at disturbance time T3
Constant (CC) regime
at disturbance time T3
water
(W)
Skin
mucus
(M)
Gut
(G)
1.5
1
0.5
0
d = 0.2
Ctrl.MT3
Ctrl.WT3
Ctrl.GT3
CV.WT3
CV.MT3
CV.GT3
CC.WT3
CC.MT3
CC.GT3
metaNMDS plot of Microbial Profiles
(p−value 0.001)

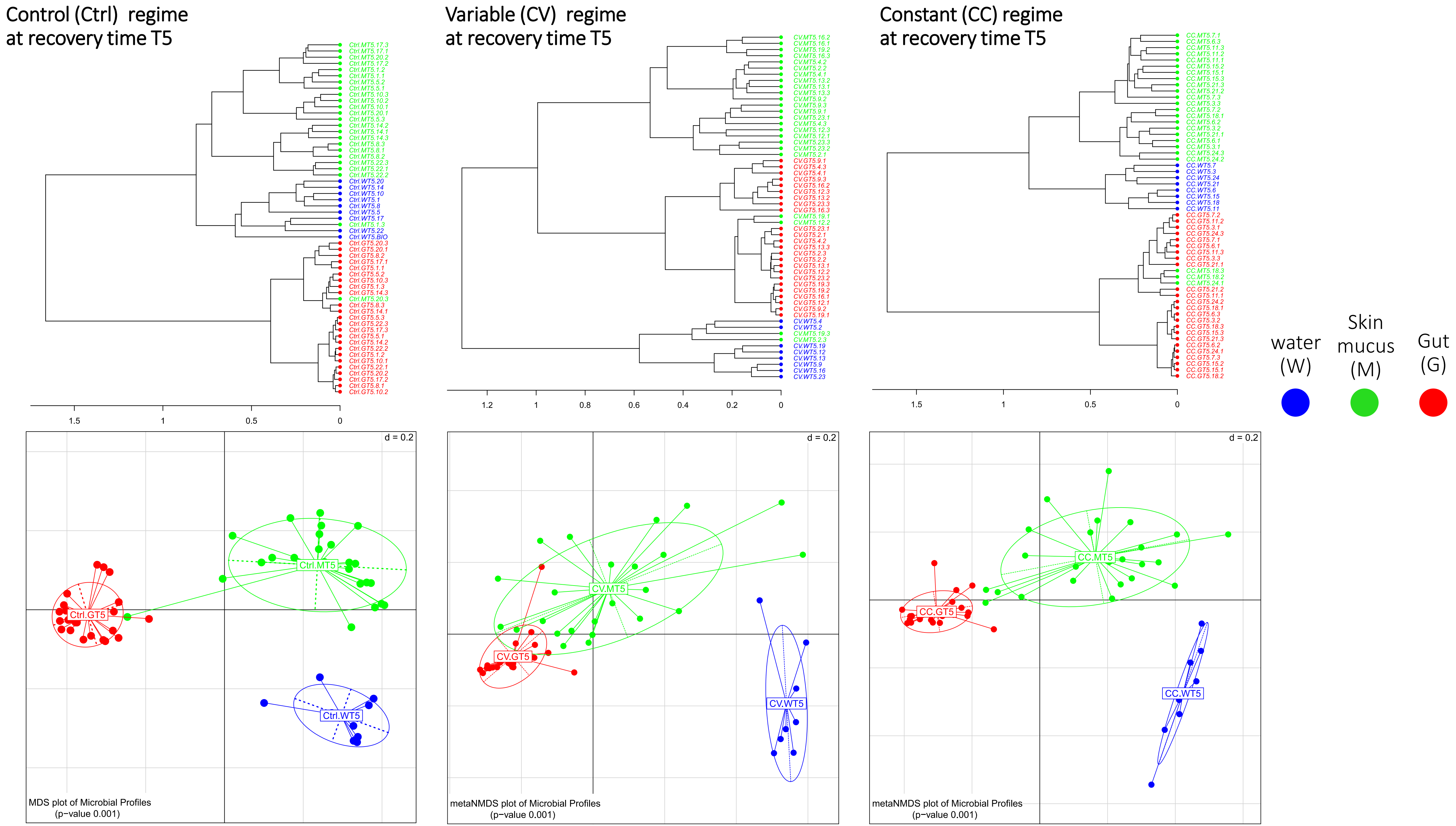

Control (Ctrl) regime
at recovery time T5
Variable (CV) regime
at recovery time T5
Constant (CC) regime
at recovery time T5
water
(W)
Skin
mucus
(M)
Gut
(G)
Ctrl.GT5
Ctrl.MT5
Ctrl.WT5
CV.GT5
CV.MT5
CV.WT5
CC.GT5
CC.MT5
CC.WT5
d = 0.2
MDS plot of Microbial Profiles
(p−value 0.001)
metaNMDS plot of Microbial Profiles
(p−value 0.001)
metaNMDS plot of Microbial Profiles
(p−value 0.001)
